# Dysregulation of the vascular endothelial growth factor and semaphorin ligand-receptor families in prostate cancer metastasis

**DOI:** 10.1186/s12918-015-0201-z

**Published:** 2015-09-04

**Authors:** R. Joseph Bender, Feilim Mac Gabhann

**Affiliations:** Department of Biomedical Engineering, Johns Hopkins University, Baltimore, MD 21218 USA; Institute for Computational Medicine, Johns Hopkins University, Baltimore, MD 21218 USA

## Abstract

**Background:**

The vascular endothelial growth factor (VEGF) family is central to cancer angiogenesis. However, targeting VEGF as an anti-cancer therapeutic approach has shown success for some tumor types but not others. Here we examine the expression of the expanded VEGF family in prostate cancer, including the Semaphorin (Sema) family members that compete with VEGFs for Neuropilin binding and can themselves have pro- or anti-angiogenic activity.

**Results:**

First, we used multivariate statistical methods, including partial least squares and clustering, to examine VEGF/Sema gene expression variability in previously published prostate cancer microarray datasets. We show that unlike some cancers, such as kidney cancer, primary prostate cancer is characterized by both a down-regulation of the pro-angiogenic members of the VEGF family and a down-regulation of anti-angiogenic members of the Sema family. We found pro-lymphangiogenic signatures, including the genes encoding VEGFC and VEGFD, associated with primary tumors that ultimately became aggressive. In contrast to primary prostate tumors, prostate cancer metastases showed increased expression of key pro-angiogenic VEGF family members and further repression of anti-angiogenic class III Sema family members. Given the lack of success of VEGF-targeting molecules so far in prostate cancer, this suggests that the reduction in anti-angiogenic Sema signaling may potentiate VEGF signaling and even promote resistance to VEGF-targeting therapies. Inhibition of the VEGF ‘accelerator’ may need to be accompanied by promotion of the Sema ‘brake’ to block cancer angiogenesis. To leverage our mechanistic understanding, and to link multigene expression changes to outcomes, we performed individualized computational simulations of competitive VEGF and Sema receptor binding across many tumor samples. The simulations suggest that loss of Sema expression promotes angiogenesis by lowering plexin signaling, not by potentiating VEGF signaling via relaxation of competition.

**Conclusions:**

The combined analysis of bioinformatic data with computational modeling of ligand-receptor interactions demonstrated that enhancement of angiogenesis in prostate cancer metastases may occur through two different routes: elevation of VEGFA and reduction of class 3 Semaphorins. Therapeutic inhibition of angiogenesis in metastatic prostate cancer should account for both of these routes.

**Electronic supplementary material:**

The online version of this article (doi:10.1186/s12918-015-0201-z) contains supplementary material, which is available to authorized users.

## Background

The vascular endothelial growth factor (VEGF) family plays an important role in promoting tumor angiogenesis, making it an attractive target in the development of cancer therapies. Therapies targeting VEGF family proteins have had success in several cancer types: bevacizumab in colorectal [1], lung [2], brain [3], and kidney [4]; aflibercept in colorectal [5]; sorafenib in kidney and liver; sunitinib in gastrointestinal stromal, kidney, and pancreatic neuroendocrine; pazopanib for kidney and soft tissue sarcoma (www.cancer.gov). However, overall survival in prostate cancer has not been improved by bevacizumab despite evidence for the importance of angiogenesis in this disease [6]. Bevacizumab and aflibercept treatment improves progression-free survival in patients with metastatic castration-resistant prostate cancer (CRPC) but has no effect on overall survival [7, 8], a pattern also observed in anti-VEGF clinical trials in breast [9] and ovarian [10] cancers. One possible reason for the failure of clinical trials is a lack of understanding of who will respond best to angiogenesis inhibitors. Better predictive biomarkers will allow for patient selection so that patients will only be treated with VEGF inhibitors if their tumors are sensitive to those inhibitors.

Several difficulties need to be overcome in developing predictive biomarkers for angiogenesis inhibitors. For example, the protein interactions within the VEGF pathway are complex: there are five genes (VEGFA, VEGFB, VEGFC, VEGFD, and PlGF) encoding ligands that bind with differing affinities to three receptor tyrosine kinases (VEGFR1, VEGFR2, and VEGFR3) and two neuropilin co-receptors (NRP1 and NRP2). The VEGF receptors can form complexes with the neuropilin co-receptors, altering both effective ligand affinities and downstream signaling outcomes [11]. An additional family of proteins, the semaphorins, also bind to neuropilins and inhibit angiogenesis, potentially through competition with VEGF for neuropilin binding sites [12]. The semaphorins also induce their own signaling by binding to members of the plexin family; thus the semaphorin/plexin family can modulate angiogenesis by (a) regulating VEGF signaling through modulation of neuropilin availability, and (b) direct anti-angiogenic signaling through plexins. This makes it necessary to consider all of these families when studying VEGF signaling. We previously observed that the aggressive triple-negative form of breast cancer is associated with simultaneous up-regulation of VEGFA and down-regulation of several class 3 semaphorins [13], both of which would be expected to increase angiogenesis. Significant variation in angiogenesis-related genes within the triple-negative subtype is also observed [13, 14]. In this study we will determine whether there are any associations between VEGF/Sema gene expression and prostate cancer prognostic factors.

Development of prostate cancer biomarkers has focused on two key questions: (a) whether localized tumors will become aggressive or remain indolent; and (b) treatment selection for metastatic CRPC. For the former, gene expression-based biomarkers have been developed to predict whether a localized tumor will become aggressive. These multicomponent biomarkers include Oncotype Dx [15], Decipher [16], and others [17–21], and are often used in conjunction with clinical disease markers such as Gleason score and pathological stage. Predictive biomarkers of treatment response to metastatic tumors that have progressed beyond androgen deprivation therapy have received less attention, but the molecular alterations present in such tumors have been studied [22, 23].

Here we consider expression of VEGF and semaphorin ligands and receptors across prostate cancer datasets. We compare the expression of these genes to biomarkers of indolent vs. aggressive primary tumors, and examine the variability of expression of these genes in metastatic tumors to propose biomarkers for selecting CRPC patients who may be sensitive to drugs that inhibit angiogenesis. We also apply a novel technique incorporating gene expression variability into computational models of growth factor pharmacodynamics, in order to predict the activation of VEGF and Sema receptors across a population of prostate cancer patients. Thus, we use molecular mechanism as well as high-throughput data to improve prediction and insight.

## Methods

A flowchart of the overall procedure of this study is in Additional file 1: Figure S1.

### Data

The datasets used in this study (Additional file 1: Table S1), were obtained from the TCGA website (http://cancergenome.nih.gov/) or from the National Center for Biotechnology Information (NCBI) Gene Expression Omnibus (GEO) database (http://www.ncbi.nlm.nih.gov/geo/). The majority of samples were from primary, untreated tumors, but some datasets also included normal prostate tissue samples and tumors that had metastasized from the prostate to various locations in the body. The data for these samples were included in the analysis, in separate groups distinct from the primary tumors. Outcome data (time until death) was not available, with the exception of some TCGA samples. The TCGA and GSE21034 datasets included biochemical recurrence (BCR) data, with times indicating the duration between sample collection and either BCR or BCR-free follow-up.

### Comparison of prostate tissue types

To assess univariate differences in gene expression across prostate tissue types (normal tissue, primary tumors, and metastatic tumors), we performed t-tests for each gene with multiple testing corrections. We also used partial least squares discriminant analysis (PLS-DA) to find multivariate patterns that differentiated the tissue types. Significance of the PLS-DA models was determined from the classification error after cross-validation. More details on these methods can be found in the supplementary information.

**Fig. 1:**
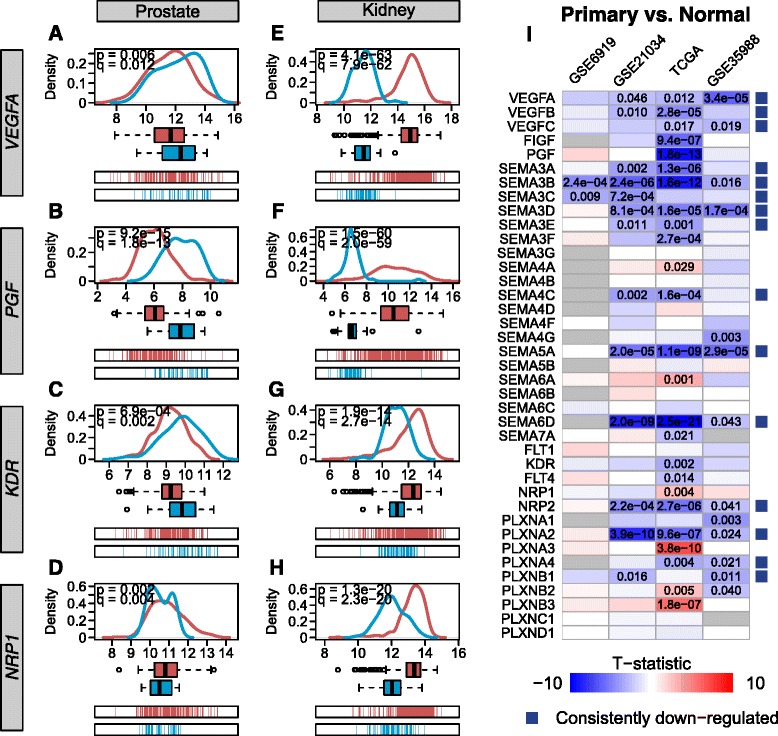
Down-regulation of pro- and anti-angiogenic ligands in primary prostate tumors. **a**-**d**: *VEGFA* (**a**), *PGF* (**b**), and the receptor gene *KDR* (**c**) are expressed at lower levels in prostate tumors than in normal prostate tissue in the TCGA dataset, as shown by the density plots (*top*), box plots (*middle*), and spike plots (*bottom*). The co-receptor gene *NRP1* (**d**) is expressed at slightly higher levels in tumors but has a similar overall distribution to normal tissues. **e**-**h**: This contrasts with the TCGA renal cell carcinoma (kidney) dataset, where *VEGFA* (**e**), *PGF* (**f**), *KDR* (**g**), and *NRP1* (**h**) are heavily up-regulated in tumors. **i** *VEGFA* down-regulation in primary prostate cancer is observed across TCGA and microarray datasets, as is consistent down-regulation of class three semaphorins. The number in the boxes indicates the two-tailed *t*-test p-value after multiple testing correction with the Benjamini-Hochberg procedure. Only comparisons with corrected p-values less than 0.05 are displayed. The colors of the boxes indicate the magnitude of the *t*-statistic. The blue boxes to the right of the rows indicate that a gene is significantly down-regulated in two or more datasets with no significant differences in other datasets

We used three types of plots to show differences in gene expression between tissue types (e.g. Fig. 1a-d and Additional file 1: Figures S2-S5): density plots show differences in the shapes of the distributions, with the densities estimated using the R function *density* with default kernel bandwidth; box plots show the range of variation with statistics such as the median and quartiles; and spike plots showed the individual points to emphasize the range.

### Prognostic significance of PLS-DA biomarkers

Data for time to follow-up or biochemical recurrence (BCR) was used to analyze survival of patients in distinct clinical or PLS-DA-derived groups. Log rank tests were used to determine differences in the Kaplan-Meier survival estimates between two classes, and Cox proportional hazard models were used to compare the effects of multiple continuous variables. More details on survival analysis methods can be found in the supplementary information (Additional file 1: Supplementary Methods).

### Comparison of multiple tumor samples from single patients

To assess the similarity of tumors from different metastatic sites within the same patient, we scaled the metastatic tumor data in GSE38241 by subtracting the mean expression of each gene in the normal samples. Then we grouped the metastasis samples using *K*-means clustering. Consensus clustering methods were applied to determine the number of clusters. More details on clustering methods can be found in the supplementary information (Additional file 1: Supplementary Methods).

### Other gene expression-based biomarkers

We also analyzed other biomarkers (Additional file 1: Table S2) using the same methodology just described. Some were based on commercially available prostate cancer diagnostics tests, such as Oncotype Dx and Decipher. In these cases, we used the genes in the diagnostic test in PLS-DA models, rather than the proprietary algorithms.

### Simulation of VEGF/Sema binding

Binding of VEGF ligands and class 3 semaphorin ligands to their receptors was modeled as a system of coupled nonlinear ordinary differential equations. The model consisted of a tumor compartment with two cell types, tumor and endothelial, similar to models we have developed previously for the VEGF family [24]. Receptors were present on both cell types and were able to bind the ligands present in the interstitial space. Tumor cell protein production rates were varied from nominal values using gene expression data from dataset GSE35988. More details on the computational model can be found in Additional file 2, with parameter values given in Additional file 2: Tables S8-S12. Schematics of the reactions between molecules and molecular complexes are shown in Additional file 2: Figure S13, and general characteristics of the model output are given in Additional file 2: Figures S14 and S15.

## Results

### Primary prostate tumor VEGF/Sema alterations

To examine why prostate cancer is less susceptible to VEGF inhibition, we first compared gene expression between primary prostate tumors and a cancer type that typically does respond to VEGF inhibitors, renal cell carcinoma [25]. Using the TCGA RNA-Seq dataset, we found that while two key pro-angiogenic ligands, *VEGFA* and *PGF*, were up-regulated in renal cell carcinoma, they were down-regulated in primary prostate adenocarcinoma (Fig. 1a, b for prostate, Fig. 1e, f for kidney). This could indicate a lack of VEGF signaling for VEGF inhibitors to target, making attempts at targeting the VEGF pathway in prostate cancer futile. This pattern was observed for other VEGF ligands: these genes were down-regulated or unchanged in prostate cancer, whereas they were up-regulated in renal cell carcinoma (Additional file 1: Figures S2-S5). Similarly, the receptors VEGFR2 (*KDR*) and NRP1 were decreased or relatively unchanged in prostate primary tumors while they were up-regulated in renal cell carcinoma (Fig. 1c, d for prostate, Fig. 1g, h for kidney). This pattern was consistent across other VEGF receptors (Additional file 1: Figures S2-S5). The up-regulation of both ligands and receptors in renal cell carcinoma clearly suggests the importance of VEGF signaling in that case. However, the up- or down-regulation of VEGF ligands and receptors in cancer types may not fully explain the response to VEGF inhibitors in prostate cancer. Subsets of tumors may have pro-angiogenic gene expression despite the anti-angiogenic pattern in the tumors as an overall group. Additionally, other pathways that interact with the VEGF pathway may affect signaling.

We considered an additional set of genes that have been demonstrated to affect VEGF signaling: the semaphorins and their plexin receptors, bringing the total number of VEGF/Sema-related genes under consideration to 39. We expanded our comparison of normal prostate tissue and primary prostate tumors to include multiple microarray datasets in addition to the TCGA dataset (Additional file 1: Table S1, note that only GSE6919, GSE21034, TCGA, and GSE35988 contain both normal prostate tissue and primary prostate tumors). For primary tumor versus normal tissue comparisons, we measured differences in gene expression using a two-tailed *t*-test with multiple testing correction using the Benjamini-Hochberg procedure. We found that in addition to VEGF ligands, many semaphorins were also down-regulated in prostate cancer (Fig. 1i). In particular, class 3 semaphorins, which have potential anti-angiogenic effects due to their ability to compete with VEGF for neuropilin binding, were down-regulated. Not all gene expression changes were consistent across all datasets; ones that were significant (defined as q-value less than 0.05) in two or more datasets were marked with blue or red boxes to the right. The subset of genes consistently down-regulated in primary tumors included three out of five VEGF ligands (*VEGFA*, *VEGFB*, and *VEGFC*) and five out of seven class 3 semaphorins (*SEMA3A*, *SEMA3B*, *SEMA3C*, *SEMA3D*, and *SEMA3E*). These results made the overall impact on angiogenesis unclear as both pro-angiogenic VEGF signals and anti-angiogenic semaphorin signals were reduced in primary tumors.

### A pro-lymphangiogenic gene expression signature is associated with aggressive primary tumors

**Fig. 2:**
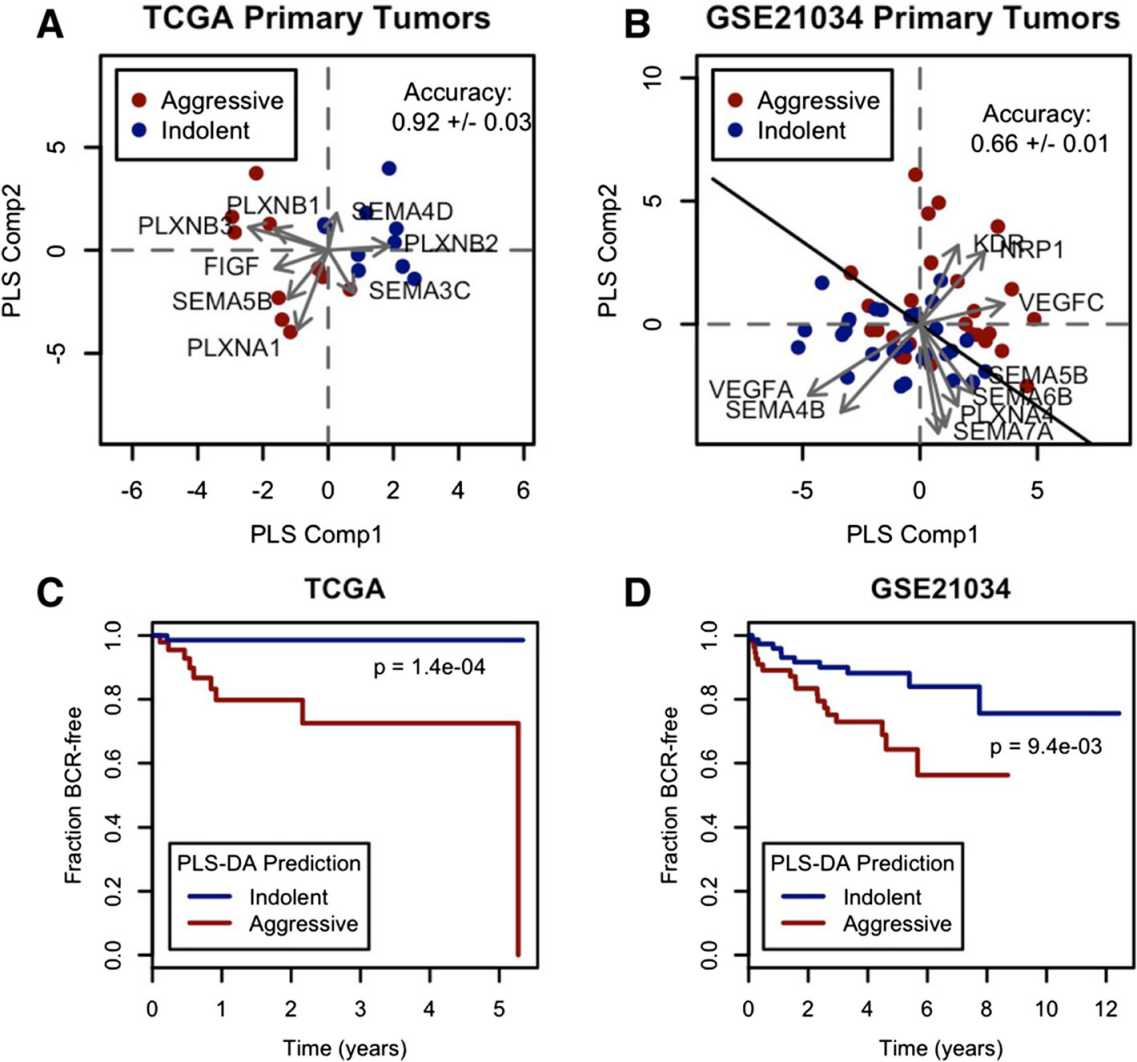
VEGF/Sema expression signatures predicting biochemical recurrence (BCR). **a**-**b**: PLS-DA scores/loadings plots for the TCGA (**a**) and GSE21034 (**b**) datasets. The training accuracies and the discriminant line separating the two classes are displayed **c**-**d**: Survival curves show prognostic significance of VEGF/Sema signatures in the TCGA (**c**) and GSE21034 (**d**) datasets. The p-values are from log rank tests of the Kaplan-Meier survival estimators for each group

To assess whether some subsets of primary prostate tumors may have differing potential benefits from VEGF signaling inhibitors, we used partial least squares discriminant analysis (PLS-DA), a multivariate algorithm that allowed us to simultaneously consider both the pattern of VEGF/Sema gene expression and effects on an output variable. As an output, we used a binary variable indicating whether biochemical recurrence (BCR) eventually occurred. Data for BCR/follow-up times were available in the TCGA and GSE21034 datasets, with 10 and 27 BCR events, respectively (Additional file 1: Table S3). Since BCR-negative samples with short follow-up times may eventually undergo recurrence, we used only the ten and 27 BCR-negative samples with the longest follow-up times in the TCGA and GSE21034 datasets, respectively. These groups of indolent samples had follow-up times greater than 3.3 years in the TCGA dataset and greater than 5.2 years in the GSE21034 dataset. These minimum indolent follow-up times were greater than all but one of the times at which BCR occurred in the TCGA dataset and all but three in the GSE21034 dataset. The TCGA PLS-DA model was effective in differentiating aggressive and indolent tumors, with a 92 ± 3 % training accuracy (Fig. 2a). The GSE21034 PLS-DA model, on the other hand, was less effective with only a 66 ± 1 % training accuracy (Fig. 2b). This discrepancy in performance between TCGA and GSE21034 datasets was seen for other biomarkers meant to distinguish aggressive from indolent tumors (Additional file 1: Figure S7). Notably, the VEGF/Sema PLS-DA model (and PLS-DA models based on the genes from other biomarkers) lost some of its predictive ability when correcting for Gleason score, but still was significantly prognostic (Additional file 1: Figure S8). ROC curves from leave-one-out cross-validation barely deviated from a 45-degree line (Additional file 1: Figure S6), indicating that these models would likely be ineffective in distinguishing aggressive and indolent tumors in other datasets. ROC curves for PLS-DA models trained with the 478 genes of the “angiome”, a set of angiogenesis-related genes [26], improved but were still fairly poor: the AUCs for TCGA and GSE21034 were 0.72 and 0.66, respectively (Additional file 1: Table S6). The 1,233 genes of the extended angiome [26] also led to poor PLS-DA models, with AUCs of 0.74 and 0.69 (Additional file 1: Table S7). Nonetheless, the PLS-DA models provided gene expression signatures with potential prognostic significance: when the PLS discriminant scores were used as predictive variables in Kaplan-Meier survival analysis, aggressive and indolent tumors had significantly different outcomes (Fig. 2c, d).

The PLS-DA models also yielded information regarding patterns of VEGF/Sema expression associated with aggressive prostate tumors. The gene-specific loadings vectors (arrows in Fig. 2a, b) are the projections of each gene onto the latent variable space, and show the extent to which different genes contribute to the variability in the response variable. The loadings revealed that the association of VEGF/Sema genes with aggressiveness was different between the two datasets. For the TCGA dataset, aggressive tumors were associated with high expression of *FIGF* (VEGFD), *NRP2*, *PLXNA1*, *PLXNB1*, *PLXNB3*, and *SEMA5B* and low expression of *PLXNB2* and *SEMA4D*. The first two of these genes, *FIGF* (VEGFD) and *NRP2*, mediate pro-lymphangiogenesis signals. The process of lymphangiogenesis may provide tumors with a route by which to escape their tissue of origin into the bloodstream [27]. In the GSE21034 model, high expression of *VEGFC*, *KDR,* and *NRP1* and low expression of *VEGFA* and *SEMA4B* were associated with aggressive tumors. Although this signature was different from the one found in the TCGA dataset, the high expression of *VEGFC* also suggested a potential for lymphangiogenic activity in aggressive primary tumors.

### Metastatic prostate tumors are associated with a pro-angiogenic signature

**Fig. 3:**
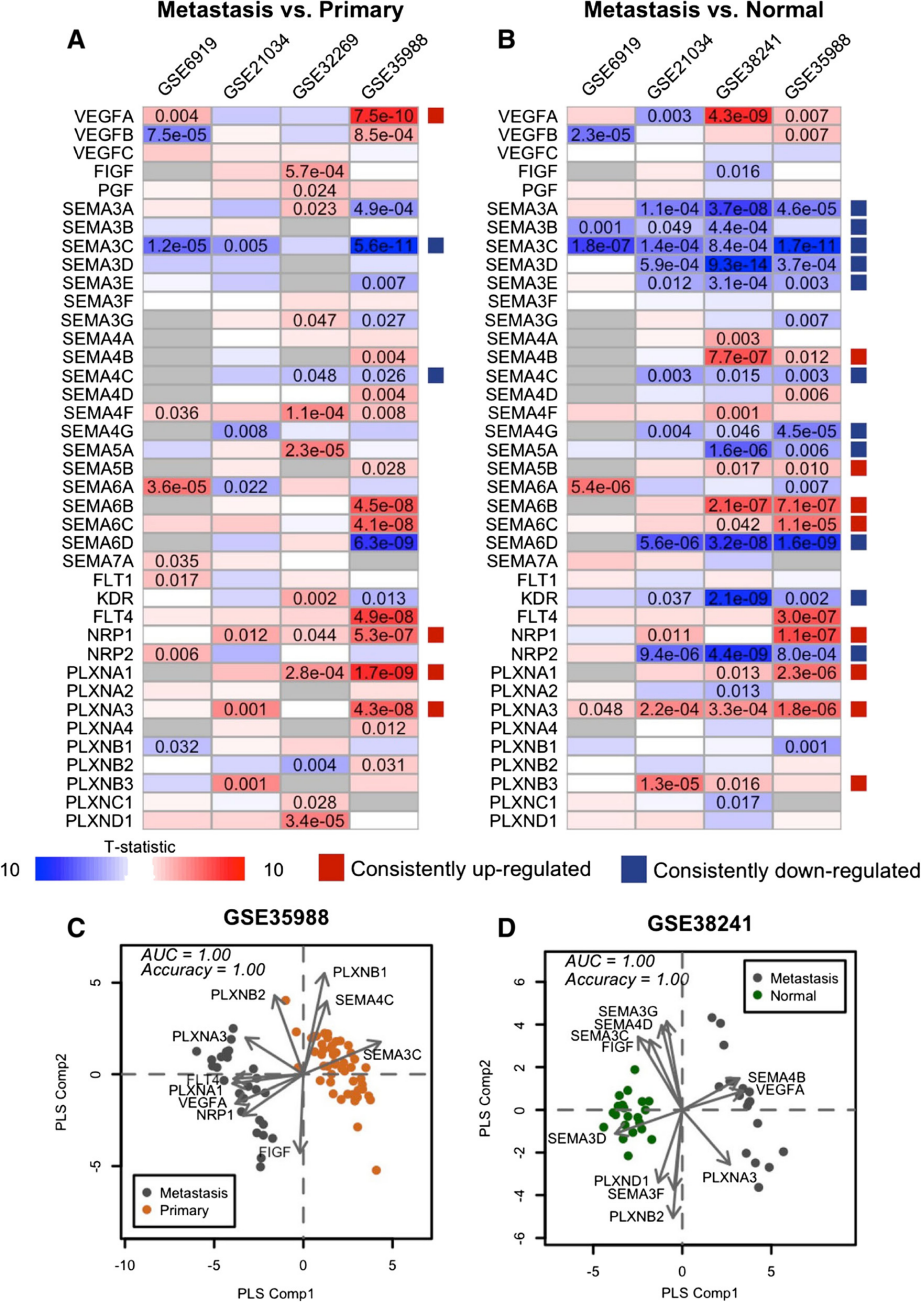
Pro-angiogenic VEGF/Sema gene expression in prostate cancer metastasis. **a**-**b**: A greater number of VEGF/Sema genes have recurrent expression alterations when comparing metastases to normal samples (**b**) than to primary tumor samples (**a**). The p-values are displayed according to the same criteria as in Fig. 1, and red and blue boxes on the right hand sides of panels **a** and **b** indicate recurrent up- and down-regulation, respectively. **c**-**d**: PLS-DA scores plots show separation of metastases and primary tumors in GSE35988 (**c**) and of metastases and normal tissues in GSE38241 (**d**). Each dot represents a sample with colors as indicated. Arrows correspond to gene loadings in the PLS-DA models, with the names of the genes displayed in the vicinity of the arrowhead. Only the genes with the largest magnitude loadings vectors are displayed. Accuracy refers to the accuracy of the LOOCV predictions; AUC refers to the area under the curve of the LOOCV ROC curve. In both cases, values of one indicate perfect prediction

While primary tumors are often treated with surgery and radiation, targeted therapeutics such as VEGF inhibitors are used more often in metastatic disease. Therefore, we next considered VEGF/Sema gene expression in datasets with metastatic tumors (Additional file 1: Table S1, note that only GSE6919, GSE21034, GSE32269, and GSE35988 contain both primary and metastatic tumors, while only GSE6919, GSE21034, GSE38241, and GSE35988 contain both normal prostate tissue and metastatic tumors). In contrast to the reduced expression of VEGF ligands in primary tumors, metastatic tumors tended to have higher expression of the major pro-angiogenic ligand, *VEGFA* (Fig. 3a, b). This ligand was up-regulated in metastases relative to primary tumors and normal tumors in the GSE6919, GSE35988, and GSE38241 datasets, but was actually down-regulated in GSE21034 and GSE32269. Notably, metastatic samples in the three datasets with up-regulated *VEGFA* were all obtained from warm autopsy programs where samples were processed rapidly upon the death of the patient. Metastatic samples in GSE32269 were from bone marrow biopsies of live patients, and no details were given regarding how metastatic samples were obtained in the GSE21034 dataset. The class 3 semaphorins were down-regulated in metastases relative to normal prostate tissue (Fig. 3b), suggesting that the loss of semaphorin expression in primary prostate tumors was maintained upon metastasis. *SEMA3C* was further down-regulated relative to primary tumors as well. Other expression alterations recurrent across datasets included up-regulation of *NRP1*, *PLXNA1*, and *PLXNA3* relative to both normal tissue and primary tumors. These three genes participate in class 3 semaphorin signaling, while only *NRP1* participates in VEGF signaling. *KDR* and *NRP2* were recurrently down-regulated in metastases relative to normal tissue but not relative to primary tumors.

We used PLS-DA to show that the VEGF/Sema gene expression alterations present in metastatic prostate tumors differed significantly from both normal tissue and primary tumors. PLS-DA models comparing metastases with primary tumors in the GSE35988 dataset and with normal tissue in the GSE38241 dataset led to large separation between the PLS scores of the two classes (Fig. 3c, d and Additional file 1: Figure S9). The leave-one-out cross-validation accuracy was 100 % for both of these comparisons.

### VEGF/Sema alterations are consistent across multiple metastases within individual patients

**Fig. 4:**
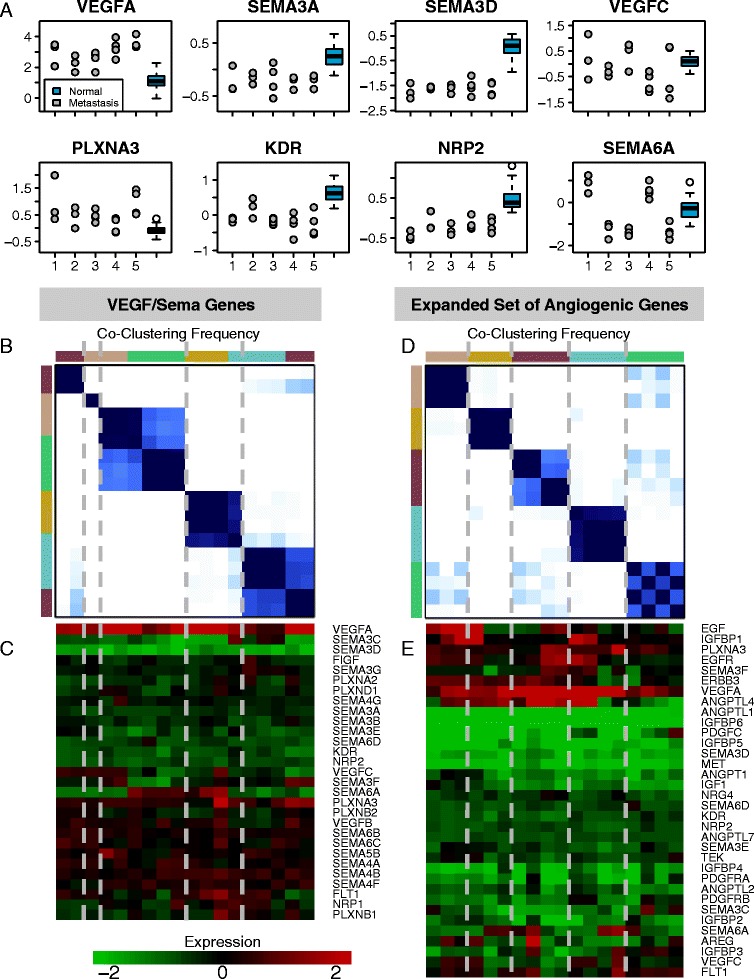
Intra-patient variability of metastases is low relative to inter-patient variability. **a** Many genes have consistent alterations in metastatic samples from the same patient while variable expression patterns between patients are more common. Blue box plots indicate the range of expression in the 21 normal samples, with the upper and lower ends of the boxes corresponding to the third and first quartiles of the data, respectively. The numbers along the *x*-axis indicate to which patient the dots above correspond. **b** Consensus *K*-means clustering of GSE38241 metastases according to VEGF/Sema expression shows a consistent co-clustering pattern for 5 clusters. Dark blue indicates a high frequency of co-clustering between consensus runs, while white indicates no co-clustering. Colors at the top and left indicate the patient from which each metastatic sample was taken. **c** Heatmap of gene expression across 18 GSE38241 metastases. The mean expression of each gene in the normal prostate tissue samples is subtracted from the expression of each gene in the metastases so that the heatmap shows up/downregulation relative to normal. Only the most variable of the VEGF/Sema genes are displayed. Dashed lines separate clusters, and colors at the top correspond to patients as in **b**. **d**-**e**: As for **b** and **c**, but with the set of 85 angiogenesis-related genes

The metastasis samples in the GSE38241 dataset included three to four metastases per patient from five different patients, providing the opportunity to analyze VEGF/Sema gene expression in both inter- and intra-patient contexts. We observed a high degree of consistency between metastases from the same patient, with *VEGFA* consistently up-regulated and several class 3 semaphorins, *KDR*, and *NRP2* consistently down-regulated (Fig. 4a; the numbers 16, 17, 21, 22, and 30 on the *x*-axis correspond to different patients). Some genes that were not significantly altered when comparing all metastases to normal samples did show patient-specific alterations: *VEGFC* and *SEMA6A* were up-regulated in some metastases and down-regulated in others. *SEMA6A* was consistent within patients, while *VEGFC* was not.

To assess the overall consistency of metastases from individual patients, we performed consensus *K*-means clustering of the metastatic samples and compared the clusters to the patients of origin. Repeating the clustering on random subsets of the data yielded a consensus matrix with clear separations of the groups when the number of clusters was five (Fig. 4b, c and Additional file 1: Figure S10). This did not perfectly separate the patients, but there was a significant association between clusters and patients (χ^2^ test *p* = 0.001). We also found that if we expanded our set of genes beyond VEGF/Sema to include other ligands and receptors important in angiogenesis (specifically, the EGF/ErbB family, HGF/Met, the Ang/Tie family, the PDGF/PDGFR family, and the IGF/IGFR family), we obtained consensus *K*-means clusters that resulted in perfect separation of the patients into clusters (Fig. 4d, e). This supports the hypothesis that, although signaling pathways that contribute to angiogenesis may vary from patient to patient, the multiple metastases that can arise from a single prostate tumor would likely all respond (or not respond) to the same or similar therapies. That this broader set of receptor tyrosine kinases and their associated ligands improves patient stratification is consistent with the need to understand a broader angiome beyond VEGF.

### Computational modeling of the VEGF/Sema pathway stratifies patients

To further analyze the contributions of gene expression changes to angiogenesis-related signaling, we developed a computational model consisting of ordinary differential equations (ODEs) describing the ligand-receptor binding kinetics of the five VEGF ligands and seven class 3 Semaphorins. A detailed model is essential, given the high inter-individual variability in gene expression, and given that predicting the outcome on signaling of simultaneous changes in expression of multiple genes encoding competing ligands and receptors becomes difficult. For a more detailed description of the model development, see *Methods* and Additional file 2. A key component of the model is that it incorporates both tumor cells and tumor endothelial cells, and these cells can express both the ligands and receptors of the VEGF and Sema pathways. Instead of using an ‘average’ model, the tumor cell ligand and receptor production rates were varied based on the gene expression data in the GSE35988 dataset, which included normal prostate, primary prostate tumors, and metastatic tumors. This enabled us to run many simulations – one for each individual – and to predict the amounts of VEGF and Semaphorin signaling complexes in a patient-specific manner.

**Fig. 5:**
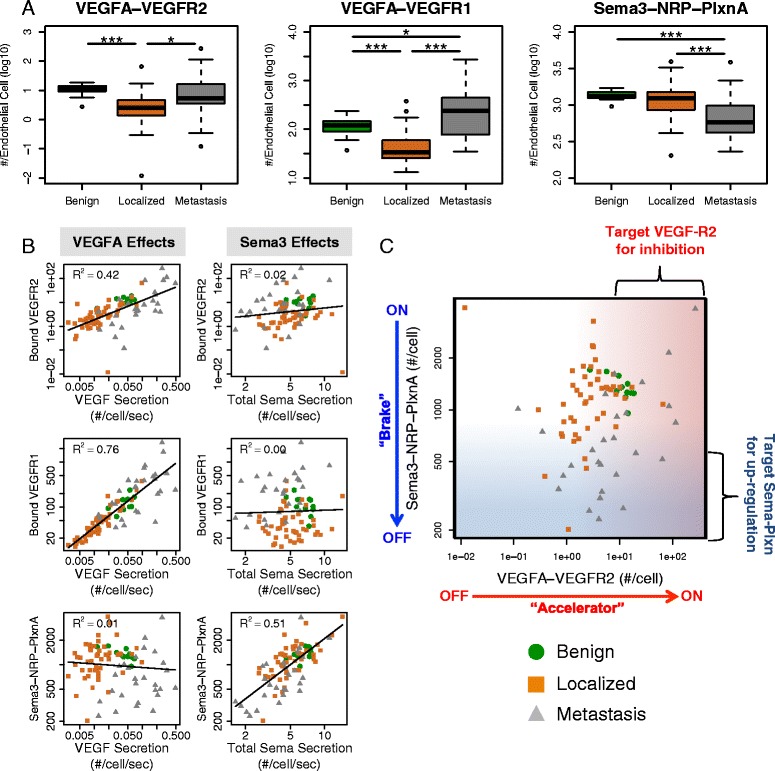
Simulated VEGF/Sema ligand-receptor binding on endothelial cells. **a** VEGFR2 (*left*), VEGFR1 (*middle*), and Sema3-NRP1 (*right*) binding in benign prostate (*n* = 12), primary tumors (*n* = 49) and metastatic tumors (*n* = 27) in the GSE35988 dataset. The p-values are marked as follows: a single * indicates *p* < 0.05, a double ** indicates *p* < 0.01, and a triple *** indicates *p* < 0.001. **b** VEGF secretion and total Sema3 secretion most strongly affect their respective ligand-receptor complexes although weak competitive effects are observed. Lines represent least squares fits of the log-transformed simulated receptor binding data to the gene expression data. R^2^ values represent the proportion of variance explained by the least squares fit. **c** Scatter plots of simulated VEGFA-VEGFR2 and Sema3-NRP-PlxnA across tissue types show that only fraction of the metastatic samples fall into the expected anti-VEGFA responsive region, i.e. high VEGFA-VEGFR2 signaling and low Sema3-NRP-PlxnA signaling. Gradients correlate with expected favorability for angiogenesis: darker red for higher VEGFA-VEGFR2 and darker blue for lower Sema3-NRP-PlxnA. Colors indicate tissue type: Benign (*green*); Localized (*orange*); Metastasis (*gray*)

The results of simulating many individuals with tumors were divided into groups (benign, localized, metastatic) and the distribution of predicted signaling outputs across the population are calculated. Binding of the two major isoforms of VEGFA (VEGF_165_ and VEGF_121_) to VEGFR-2 and VEGFR-1 on endothelial cells was lowest in primary (localized) tumors, whereas the collective binding of the class 3 Semaphorins to endothelial plexins decreased in primary tumors relative to normal tissue and decreased further in metastatic tumors (Fig. 5a). The receptor binding trends closely followed ligand expression levels, with *VEGFA* expression being the predominant factor driving VEGFR-1/2 binding, while the total Sema3 expression accounted for most of the variation in Sema3-NRP-PlxnA binding (Fig. 5b). The receptor binding profiles on tumor cells (Additional file 1: Figure S12) were similar to those on endothelial cells. These results indicated that primary tumors are associated with both pro-angiogenic (decreased Sema3-NRP-PlxnA binding) and anti-angiogenic (decreased VEGFR-2 binding) alterations, making it difficult to predict whether primary tumors would benefit from therapies that inhibit VEGF signaling. On the other hand, the alterations in metastatic tumors were all pro-angiogenic: VEGFR-2 binding was higher and Sema3-NRP-PlxnA binding was lower. The range of VEGFR-2 binding was highest in metastatic samples, going beyond both the low and high ends of the range of normal samples. This suggested an important role for patient selection in the use of anti-angiogenic therapies, as only the patients with high baseline VEGFR-2 signaling would be expected to respond.

Aside from anti-angiogenic signaling initiated by Sema3 binding to neuropilins and plexins, Sema3s also may inhibit angiogenesis by competitively displacing VEGF from neuropilins. In our model, we found only a weak competitive effect: the least squares fits for the non binding ligand-receptor pairs (VEGFA effects on Sema3-NRP-PlxnA and Sema3 effects on VEGFA-VEGFR-2 in Fig. 5b) had slight negative slopes, but the least squares models explained a very small proportion of the overall variance. This was due to the fact that the amount of NRP1 and NRP2 present in ligand-containing signaling complexes was low relative to the total amount of neuropilins present on endothelial cells. If the amount of endothelial NRP1 and/or NRP2 were lower or the ligand secretion rates were higher, competition would be expected to have more impact. Thus, although direct VEGF-Sema competition for neuropilin is included in the model, the quantitative impact of the competition is predicted to be small.

Our simulation results suggest that determining patients with sensitivity to anti-angiogenic therapies would require biomarkers consisting of multiple predictor variables. Each individual tumor has a different predicted level of VEGFA-VEGFR-2 and low Sema3-NRP-PlxnA activity (Fig. 5c). The red and blue gradients, and the arrows on the axes, indicate direction of increasing pro-angiogenic signaling for VEGFR2 (accelerator ‘ON’) and PlxnA1 (brake ‘OFF’), respectively. Metastatic samples predominantly have high simulated VEGFA-VEGFR-2 and low Sema3-NRP-PlxnA (lower right quadrant of Fig. 5c: accelerator ‘ON’, brake ‘OFF’), and would be expected to benefit the most from VEGF-targeting therapies. However, this is not true of all metastatic tumors; a minority of patients with high VEGFA-VEGFR-2 also have high Sema3-NRP-PlxnA, possibly negating any clinical benefit of an anti-VEGFA agent. That combination is also predicted to be the most common for the benign tissues (Fig. 5c, green symbols). There are also a subset of metastatic tumors predicted to have low VEGFA-VEGFR2 signaling. This heterogeneity amongst the metastatic tumors reinforces the need to bring an individualized understanding to therapeutic selection. For those with localized disease (Fig. 5c, orange symbols) the characteristic signaling state is low VEGFA-VEGFR2 and high Sema-Plexin (upper left quadrant of Fig. 5c: accelerator ‘OFF’, brake ‘ON’), correlating with low angiogenesis potential and no metastases. Further analysis of this model and availability of both gene expression and clinical anti-VEGF outcome data will allow us to develop simulation-based biomarkers.

## Discussion

As with many types of cancer, angiogenesis may enable prostate cancer growth and progression. Tumor vessels tend to be more irregular than normal prostate vessels [6, 28], and higher microvessel density is associated with higher tumor stage [29, 30]. Some studies have reported a reduction in tumor microvessel density relative to normal prostate tissue, but even in these studies it is noted that ’hotspots” exist i.e. regions with locally high microvessel densities [28]. Prostate tumors are known to have intra-tumoral heterogeneity [31, 32], raising the possibility that these hotspots correspond to regions with a pro-angiogenic genomic signature. Multiple molecular regulators of angiogenesis could be responsible for the tumor-associated changes in the microvasculature. The predominant pro-angiogenic factor, VEGFA, is elevated in the plasma of patients with metastatic prostate cancer, but not in patients with localized primary prostate tumors [33–37] (Additional file 1: Table S4). This does not rule out a role for VEGFA in primary tumors, as immunohistochemistry has revealed increased VEGFA in primary tumors relative to benign prostate tissue, and in castration-resistant tumors relative to hormone-naïve [38]. Our analysis of previously published gene expression data in this study revealed increases in *VEGFA* expression only in metastatic tumor samples; *VEGFA* expression in primary tumors was actually decreased. Despite elevations in VEGFA in metastatic disease, most VEGF inhibitors have failed clinical trials in metastatic CRPC [7, 8, 39, 40] (Additional file 1: Table S5). One compound that has shown some promise in phase II trials, cabozantinib, also targets the Met receptor [41]. The lack of success of VEGF inhibitors suggests that other angiogenesis modulators may be involved. Therefore, we performed an analysis of VEGF-related genes and a family of potential modulators, the semaphorins, across stages of prostate cancer to gain a system-wide perspective on VEGF activity in this disease. Our methodology could be widened in the future to include additional relevant RTK families, including those most relevant to angiogenesis (Fig. 4e).

An active area of research in prostate cancer molecular biomarkers is predicting whether a primary tumor will eventually become aggressive or if it will remain indolent as indolent prostate cancers can often be left untreated and monitored. We found that a multivariate VEGF/Sema signature was associated with aggressive tumors. We used PLS-DA for distinguishing between aggressive and indolent tumors, as well as different tissue types, as it provided both effective classification and information about the correlation structure within the gene expression data. This allowed for interpretation of the expected joint effects of expression variation on VEGF signaling activity. We expected the performance of this approach to be comparable to other multivariate methods used for similar purposes, including linear discriminant analysis [18], random forests [16], and decision trees [19]. Our VEGF/Sema PLS-DA biomarkers had similar prognostic capabilities to PLS-DA biomarkers based on the genes from other prostate cancer prognostic indicators available and in development for clinical use. We used PLS-DA, as opposed to the algorithm actually used in the prognostic indicators, because the algorithms and parameters used in these indicators were proprietary. The VEGF/Sema signature of aggressiveness that we found contained several genes that promote lymphangiogenesis, including *FIGF* (VEGFD), *NRP2*, *VEGFC*, and *KDR*. This fits with previous research that suggests lymphangiogenesis plays a role in allowing primary tumors to escape their tissue of origin via the lymphatic vessels [27, 42, 43]. Inhibiting one or more of these genes could be an effective therapeutic mechanism in patients whose tumors are predicted to be aggressive, possibly as a neoadjuvant therapy prior to radical prostatectomy.

Most approaches to targeting VEGF-dependent cancer angiogenesis have relied on inhibiting VEGF or VEGFR2. This focus on blocking the accelerator of tumor angiogenesis may have overlooked a key aspect of tumor angiogenesis – the endogenous brakes provided by the Semaphorins. Analysis of metastatic tumor gene expression suggested several possible reasons for the failure of anti-angiogenic therapies in prostate cancer. As noted above, *VEGFA* expression was consistently elevated in metastases relative to normal samples, but treatment of metastatic prostate cancer with VEGF inhibitors typically fails. Reductions in class 3 semaphorins were observed, which could enhance VEGF signaling by making more NRP1 available; lower Sema3 levels could also enhance angiogenesis by removing inhibitory signals mediated by plexins (i.e. the brakes on tumor angiogenesis are removed). Our simulation data provide mechanistic insight that supports this latter case: Sema3s did not appear to alter the availability of NRP1 to VEGF, but the formation of anti-angiogenic Sema3-Plexin complexes was decreased in metastases. Thus one possible mechanism of resistance to VEGF inhibitors is reduced anti-angiogenic Sema3 signaling. With the repression of anti-angiogenic Sema signaling, blocking the accelerator may be insufficient to halt the runaway tumor vasculature; thus treatment might best be achieved, or augmented, by restoring or replacing the endogenous brakes on tumor angiogenesis.

We observed high correlation among the various metastases from each individual (Fig. 4). These multiple metastases arising from a single primary tumor may then respond similarly to particular therapies. Also, given recent findings of heterogeneity in primary tumors, it may suggest that either (a) metastases come from a selected subset of the primary tumor or (b) environments receiving and nurturing metastases may cause the metastatic tumor cells to converge. The gene expression clustering analysis in this study also presented other possible resistance mechanisms that we did not simulate. For example, *SEMA6A* was up-regulated in metastases from two patients and down-regulated in three. It has roles in angiogenesis, potentially through an interaction with VEGFR2 [44].

To move beyond the ‘average patient’ that is typically simulated by molecularly-detailed mechanistic computational models, we have integrated mechanistic simulations with high-throughput data to create a population of tumor models that can simulate variability in receptor activation and response to treatment. Our simulation results suggest that determining patients with sensitivity to anti-angiogenic therapies would require biomarkers consisting of multiple predictor variables. This is one of the most important considerations for this type of approach. Linear approaches such as PLS-DA are ultimately limited to identifying linear combinations of effects within the gene expression data. By adding the mechanism-based, quantitative, nonlinear protein-interaction network, we can generate latent variables that integrate both gene expression and mechanistic information. These predicted mechanistic latent variables may then be more predictive of the angiogenesis potential of the tumor and of the outcome of therapeutic inhibition.

Simulations of the kind presented here can be expanded to incorporate additional proteins and multiple tissue compartments to model drug pharmacokinetics and pharmacodynamics. This will be useful for developing patient-specific models capable of identifying the most appropriate molecular therapies. Therapies could include VEGF-targeting agents such as bevacizumab as well as drugs that target neuropilin, either by blocking ligand binding to neuropilin or by blocking the coupling of neuropilin to other receptors. Inclusion of semaphorins in these models allows us to analyze whether neuropilin-targeting therapies could inadvertently have a pro-angiogenic effect due to reduced semaphorin signaling. Additional semaphorins (classes 4, 5, 6, and 7) could be added as data for the kinetics becomes available. Further development of the model could also address several of the limitations of the current model. Proteolytic processing is known to alter the receptor binding and therefore the activity of VEGFC, VEGFD, and several of the class 3 Semaphorins. We neglected these effects here due to a lack of data describing the relative amounts of processed and unprocessed forms in tumors. Additionally, we have assumed that competition of VEGF and class 3 Semaphorins occurs due to the inability of both to occupy neuropilin receptors simultaneously. Further validation against published *in vitro* data may allow us to refine this assumption for specific VEGF and Semaphorin ligands; there is evidence that certain ligands (and proteolytically processed forms) are more or less able to sterically inhibit the binding of other ligands to neuropilin.

Several limitations of gene expression data may affect the conclusions of this study. Prostate tumors are typically multifocal and heterogeneous [23, 45], creating the possibility of gene expression data for a patient that does not reflect the clone that actually gives rise to aggressive disease. This is a possible explanation for the difference between the TCGA and GSE21034 datasets in the VEGF/Sema signatures found in this study to distinguish aggressive and indolent tumors. The lack of stability of mRNA leads to further issues with the measurement of mRNA expression. If appropriate protocols are not followed, degradation of mRNA can lead to inaccuracies in measurements. This is another possible explanation for the discrepancy between the TCGA and GSE21034 aggressiveness predictors, as well as for the varying changes in gene expression between metastatic samples. The expression of *VEGFA* was up-regulated in metastasis samples in GSE6919, GSE35988, and GSE38241, but not in GSE32269 or GSE21034. The three datasets with *VEGFA* up-regulation were all obtained from rapid autopsy programs of prostate cancer patients. Metastatic samples in GSE32269 were obtained from bone marrow biopsies, and the sampling protocol for GSE21034 was not specified.

Additionally, gene expression levels are not always representative of the level of the corresponding protein. Instead of equating gene expression to protein levels, we assumed that protein secretion *rates* were proportional to gene expression in our patient-specific simulations. This is a useful first approximation; further analysis could include miRNA data and other regulatory factors that would be expected to influence the rate of translation of a transcript to a protein. A final limitation is the small size of metastasis datasets. Metastatic samples are of particular interest because metastases give prostate cancer its lethality and here we show that they may have increased VEGF signaling activity. A larger rapid autopsy dataset would allow expansion of limited analysis performed here.

## Conclusions

Our analysis of gene expression data across normal prostate, primary prostate tissue, and metastatic prostate tumors revealed that while class 3 Semaphorin expression was reduced in both primary and metastatic tumors, *VEGFA* expression was elevated only in metastatic tumors. Within primary tumors, distinct clusters based on VEGF/Sema expression were not observed, but a pro-lymphangiogenic signature was associated with the likelihood of a primary tumor eventually becoming aggressive. Within metastatic tumors, alterations in VEGF/Sema expression tended to vary between patients but not between multiple metastases within a single patient.

We incorporated gene expression data into a mathematical model of the ligand-receptor interactions among all VEGF and class 3 Semaphorin ligands and their corresponding receptors. This was done by varying protein production rates based on the level of expression of the corresponding gene. At physiological ligand concentrations, no competition between VEGF and Semaphorins for Neuropilin binding was observed; instead, the level of VEGF and Semaphorin signaling complexes were independent, with some metastatic tumors exhibiting elevated VEGFA receptor binding, others exhibiting reduced Semaphorin receptor binding, and some exhibiting both. The therapeutic implications of this are that some patients may require only an anti-VEGF drug to inhibit angiogenesis, others may require only augmentation of class 3 Semaphorin signaling, while others may require both. The model also predicted that targeting Neuropilins might be problematic due to reduction in anti-angiogenesis signaling of Plexins confounding the reduction in pro-angiogenesis signaling of VEGFRs.

## Additional files

Additional file 1:
**Supplemental Methods, Figures and Tables for bioinformatic analysis. Table S1. **Datasets used in this study. **Table S2.** Gene expression-based prognostic markers. **Table S3. **Primary tumor characteristics. **Table S4. **Plasma concentrations of VEGF family members. **Table S5.** Angiogenesis inhibitor clinical trial results in prostate cancer. **Table S6.** Significant genes in the 478-gene angiome. **Table S7.** Significant genes in the 1233-gene extended angiome. **Figure S1.** Flowchart of the methods in this study. **Figure S2.** Differential expression of VEGF/Sema3 ligands and receptors in prostate cancer. **Figure S3.** Differential expression of Sema4/5/6/7 ligands and receptors in prostate cancer. **Figure S4.** Differential expression of VEGF/Sema3 ligands and receptors in renal cell carcinoma. **Figure S5.** Differential expression of Sema4/5/6/7 ligands and receptors in renal cell carcinoma. **Figure S6.** ROC curves for LOOCV of PLS-DA models of VEGF/Sema expression in aggressive and indolent tumors in the TCGA and GSE21034 datasets. **Figure S7.** Biomarker performance in TCGA and GSE21034 datasets. **Figure S8.** Cox proportional hazards modeling of the association between PLS-DA biomarkers and biochemical recurrence (BCR). **Figure S9.** PLS-DA models. **Figure S10.** Consensus *K*-means clustering. **Figure S11.** Isoform ratios of genes with alternative splicing. **Figure S12.** Tumor cell receptor binding profiles. (DOCX 6623 kb) Additional file 2:
**Computational model development, parameters and supplemental figures. Table S8.** Geometric parameters. **Table S9.** Rate constants of ligand-receptor binding reactions. **Table S10.** Rate constants of receptor coupling reactions. **Table S11.** Target free ligand concentrations. **Table S12.** Model parameters. **Figure S13.** Reactions in the VEGF/Sema model. **Figure S14.** Receptor fractional occupancies at steady-state. **Figure S15.** Effect of ligand secretion rates on receptor binding. (DOCX 5884 kb)

## Abbreviations

VEGF: Vascular endothelial growth factor
Sema: Semaphorin
PlGF: Placental growth factor
VEGFR: VEGF receptor
NRP: Neuropilin
CRPC: Castration-resistant prostate cancer
TCGA: The Cancer Genome Atlas
BCR: Biochemical recurrence
PLS-DA: Partial least squares-discriminant analysis
ROC: Receiver operating characteristic
AUC: Area under the curve
ODE: Ordinary differential equation
