# Supplementary material for: Dysregulation of the vascular endothelial growth factor and semaphorin ligand-receptor families in prostate cancer metastasis
Source: BMC Syst Biol. 2015 Sep 4;9:55. doi: 10.1186/s12918-015-0201-z (PMC4559909; doi:10.1186/s12918-015-0201-z)
Supplement: Additional file 1: — Supplemental Methods, Figures and Tables for bioinformatic analysis. Table S1. Datasets used in this study. Table S2. Gene expression-based prognostic markers. Table S3. Primary tumor characteristics. Table S4. Plasma concentrations of VEGF family members. Table S5. Angiogenesis inhibitor clinical trial results in prostate cancer. Table S6. Significant genes in the 478-gene angiome. Table S7. Significant genes in the 1233-gene extended angiome. Figure S1. Flowchart of the methods in this study. Figure S2. Differential expression of VEGF/Sema3 ligands and receptors in prostate cancer. Figure S3. Differential expression of Sema4/5/6/7 ligands and receptors in prostate cancer. Figure S4. Differential expression of VEGF/Sema3 ligands and receptors in renal cell carcinoma. Figure S5. Differential expression of Sema4/5/6/7 ligands and receptors in renal cell carcinoma. Figure S6. ROC curves for LOOCV of PLS-DA models of VEGF/Sema expression in aggressive and indolent tumors in the TCGA and GSE21034 datasets. Figure S7. Biomarker performance in TCGA and GSE21034 datasets. Figure S8. Cox proportional hazards modeling of the association between PLS-DA biomarkers and biochemical recurrence (BCR). Figure S9. PLS-DA models. Figure S10. Consensus K-means clustering. Figure S11. Isoform ratios of genes with alternative splicing. Figure S12. Tumor cell receptor binding profiles. (DOCX 6623 kb) [file 12918_2015_201_MOESM1_ESM.docx]

***Supplement 1 for***

**Dysregulation of the Vascular Endothelial Growth Factor and Semaphorin ligand-receptor families in prostate cancer metastasis**

R. Joseph Bender and Feilim Mac Gabhann

**Supplement 1: Supplemental Methods, Figures and Tables for bioinformatic analysis**

**Supplemental Methods: Statistical Methods 2-3**

**Table S1: Datasets used in this study.** 4

**Table S2: Gene expression-based prognostic markers. 5**

**Table S3: Primary tumor characteristics. 6**

**Table S4: Plasma concentrations of VEGF family members. 7**

**Table S5: Angiogenesis inhibitor clinical trial results in prostate cancer. 8**

**Table S6: Significant genes in the 478-gene angiome. 9**

**Table S7: Significant genes in the 1233-gene extended angiome. 10**

**Figure S1: Flowchart of the methods in this study. 11**

**Figure S2: Differential expression of VEGF/Sema3 ligands and receptors in prostate cancer. 12**

**Figure S3: Differential expression of Sema4/5/6/7 ligands and receptors in prostate cancer. 13**

**Figure S4: Differential expression of VEGF/Sema3 ligands and receptors in renal cell carcinoma.
 14**

**Figure S5: Differential expression of Sema4/5/6/7 ligands and receptors in renal cell carcinoma.
 15**

**Figure S6: ROC curves for LOOCV of PLS-DA models of VEGF/Sema expression in aggressive and indolent tumors in the TCGA and GSE21034 datasets. 16**

**Figure S7: Biomarker performance in TCGA and GSE21034 datasets. 17**

**Figure S8: Cox proportional hazards modeling of the association between PLS-DA biomarkers and biochemical recurrence (BCR). 18**

**Figure S9: PLS-DA models. 19**

**Figure S10: Consensus *K*-means clustering. 20**

**Figure S11: Isoform ratios of genes with alternative splicing. 21**

**Figure S12: Tumor cell receptor binding profiles. 22**

**Supplemental References 23-24**

**Supplemental Methods: Statistical methods**

*Differential expression*. Univariate gene expression differences were determined using Welch two-sample unpaired *t*-tests in R using the *t.test* function. P-values were subjected to multiple testing correction using the Benjamini-Hochberg procedure [[1](#_ENREF_1)].

*Partial least squares discriminant analysis (PLS-DA)*. To compare distinct groups, we used PLS-DA, which finds latent variables that are aligned with the direction of most co-variability between the gene expression data and the output class variable. We used the *plsda* function in the *mixOmics* package in R. The function results in a decomposition of the gene expression matrix **X** = **TP** + **E**, where **T** is the scores matrix, **P** is the loadings matrix, and **E** is a residuals matrix. The function also decomposes the output matrix **Y** = **TQ** + **F**, where **Q** is the Y-loadings matrix and **F** is a residuals matrix. A PLS weights matrix, **W**, is also returned for predicting the scores from the gene expression data, **T** = **XW**. Thus, if an output is unknown for a particular sample, the output can be predicted from the gene expression data using **Y** = **XWQ'**.

*Classifier training*. Training ~~errors~~ accuracies were determined by training a PLS-DA classifier using all samples, and then performing classification on the samples. The number of correctly ~~mis~~classified samples divided by the total number of samples was the training ~~error~~ accuracy. To find 95% confidence intervals for this estimate of the training accuracy, we used a bootstrap resampling procedure. We trained PLS-DA classifiers on 1,000 training sets generated by sampling observations from the original dataset with replacement. These 1,000 classifiers were used to predict the outcome for each observation. We averaged the accuracy of each model across all observations, then computed 95% confidence intervals on the resulting vector of accuracies. To estimate the generalization error of the PLS-DA classifiers, we used leave-one-out cross-validation (LOOCV). The generalization error was the number of misclassified samples divided by the total number of samples. The PLS-DA classification was performed n times, where n was the number of samples in the data matrix. At each iteration, one sample was held out and a classifier was trained on the data consisting of n-1 samples. Then, the expression data for the held out sample was used to predict the class of the held out sample using **Y** = **XWQ'**.

*Survival analysis*. Data for time to follow-up or biochemical recurrence (BCR) was used to analyze survival of patients in distinct clinical or PLS-DA-derived groups. We used several functions in the R *survival* package. The *survfit* function was used to compute the Kaplan-Meier estimator of survival and perform the log rank test for the difference between two discrete classes. We used the *coxph* function to fit Cox proportional hazard models to determine the relative effects of different continuous variables on survival.

*Consensus clustering*. We separated samples into clusters using the *kmeans* function in R. This function was executed 20 times to avoid local minima; the cluster membership that gave the lowest within-cluster sum of squares was the one that the *kmeans* function returned. The number of clusters to be found is pre-specified. To choose the appropriate number of clusters, consensus clustering is performed. This entails clustering 100 times on random subsets of the data. This results in a consensus matrix, which describes the co-clustering frequency of any two samples across consensus runs. Ideally, all values in this matrix will either be 1 (always in the same cluster) or 0 (never in the same cluster). The appropriate number of clusters is chosen as the value of K that has the most consensus matrix values near 1 and 0.

**Table S1: Datasets used in this study.**

| **Dataset** | **Platform** | **Tissue Types** | **Notes** | **Reference** |
| --- | --- | --- | --- | --- |
| TCGA | RNA-seq | 44 normal  176 primary | Copy number, miRNA, BCR | cancergenome.nih.gov |
| GSE21034 | Exon 1.0 Array | 29 normal  131 primary  19 metastasis | Copy number and  BCR | [[2](#_ENREF_2)] |
| GSE6919 | HG U95Av2 | 18 donor normal  63 adjacent normal  65 primary  25 metastasis | Some adjacent normal and primary tumors are paired | [[3](#_ENREF_3)] |
| GSE32269 | HG U133A | 22 primary  29 bone metastasis | Metastases from bone marrow biopsies | [[4](#_ENREF_4)] |
| GSE38241 | Agilent Whole Human Genome 4x44K | 21 normal donors  18 metastases from 5 patients | Rapid autopsies; multiple metastases per patient | [[5](#_ENREF_5)] |
| GSE35988 | Agilent Whole Human Genome 4x44K | 12 normal  49 primary  27 metastasis | Rapid autopsies | [[6](#_ENREF_6)] |

**Table S2: Gene expression-based prognostic markers.**

| **Markers** | **Company** | **Description** | **Launch Date** | **Genes^a^** |
| --- | --- | --- | --- | --- |
| Decipher | GenomeDx Biosciences | 22-gene signature to predict risk of recurrence or metastasis following radical prostatectomy | 2013 | *LASP1, IQGAP3, NFIB, S1PR4, THBS2, ANO7, PCDH7, MYBPC1, EPPK1, PBX1, NUSAP1, ZWILCH, UBE2C, CAMK2N1, RABGAP1, TNFRSF19* |
| OncotypeDx | Genomic Health | 17-gene signature to predict whether early-stage disease will remain indolent or become aggressive | May 2013 | *AZGP1, KLK2, FAM13C, FLNC, GSN, TPM2, GSTM2, TPX2, BGN, COL1A1, SFRP4, ARF1, ATP5E, CLTC, GPS1, PGK1* |
| Prolaris | Myriad Genetics | 46 cell cycle progression genes for predicting aggressiveness in multiple settings, including early-stage and post-prostatectomy | March 2010 | *FOXM1, CDC20, CDKN3, CDK1, KIF11, KIAA0101, NUSAP1, CENPF, ASPM, BUB1B, RRM2, DLGAP5, BIRC5, KIF20A, PLK1, TOP2A, TK1, PBK, ASF1B, SKA1, RAD54L, PTTG1, CDCA3, MCM10, PRC1, DTL, CEP55, RAD51, CENPM, CDCA8, ORC6, RPL38, UBA52, PSMC1, RPL4, RPS29, SLC25A3, CLTC, TXNL1, PSMA1, RPL8, MMADHC, RPL13A, PPP2CA, MRFAP1* |
| ^a^ Only genes that are present in the datasets are listed, therefore the number of genes listed may be less than the numbers in the “Description” column. | | | | |

**Table S3: Primary tumor characteristics.**

| **Dataset** |  | **TCGA** | **GSE21034** | **GSE6919** | **GSE41408** | **GSE35988** |
| --- | --- | --- | --- | --- | --- | --- |
| Gleason score | ≤6 | 11 (7%) | 78 (60%) | 19 (29%) | 23 (48%) | NA |
|  | 7 | 113 (72%) | 42 (32%) | 27 (42%) | 16 (33%) | NA |
|  | ≥8 | 33 (21%) | 11 (8%) | 19 (29%) | 9 (19%) | NA |
| BCR | yes | 10 (8%) | 27 (21%) | NA | 27 (56%) | NA |
|  | no | 118 (92%) | 104 (79%) | NA | 21 (44%) | NA |
| Median BCR time (months) |  | 6.9 | 25.5 | NA | NA | NA |

**Table S4: Plasma concentrations of VEGF family members.**

| **Protein** | **Population** | **Median (Range)** | **Reference** |
| --- | --- | --- | --- |
| VEGF-A | Healthy (n=26) | 0 (0 – 24) | [[7](#_ENREF_7)] |
|  | Healthy (n=12) | 26.5 (25-50) | [[8](#_ENREF_8)] |
|  | BPH (n=50) | 6.8 | [[9](#_ENREF_9)] |
|  | Cancer (n=50) | 13 |  |
|  | Localized tumor (n=54) | 7 (0 – 26.5) | [[7](#_ENREF_7)] |
|  | Untreated baseline (n=14) | 135 (92-177) | [[10](#_ENREF_10)] |
|  | ADT baseline (n=23) | 175 (107-245) |  |
|  | Untreated 12 weeks (n=13) | 117 (83-195) |  |
|  | ADT 12 weeks (n=22) | 146 (111-208) |  |
|  | Metastatic to bone or lymph node (n=26) | 28.5 (19.3 – 57.0) | [[7](#_ENREF_7)] |
|  | Invasive cancer (n=30) | 210 (166-360) | [[8](#_ENREF_8)] |
|  | mCRPC (n=28) | 105 +/- 101 (mean+/-SD) | [[11](#_ENREF_11)] |
| PlGF | Untreated baseline (n=14) | 12 (10-15) | [[10](#_ENREF_10)] |
|  | ADT baseline (n=23) | 13 (11-15) |  |
|  | Untreated 12 weeks (n=14) | 13 (12-14) |  |
|  | ADT 12 weeks (n=22) | 15 (13-18) |  |
| VEGF-C | BPH (n=50) | 823.7 | [[9](#_ENREF_9)] |
|  | Cancer (n=50) | 832.6 |  |
| sVEGFR-1 | Healthy (n=12) | 2250 (1500-21500) | [[8](#_ENREF_8)] |
|  | Invasive cancer (n=30) | 600 (100-4000) |  |
|  | Untreated baseline (n=14) | 81 (66-98) | [[10](#_ENREF_10)] |
|  | ADT baseline (n=23) | 66 (55-106) |  |
|  | Untreated 12 weeks (n=13) | 84 (60-102) |  |
|  | ADT 12 weeks (n=23) | 70 (55-91) |  |
| sVEGFR-2 | Untreated baseline (n=14) | 6946 (6232-8084) | [[10](#_ENREF_10)] |
|  | ADT baseline (n=23) | 6979 (5816-8882) |  |
|  | Untreated 12 weeks (n=14) | 6284 (5889-8313) |  |
|  | ADT 12 weeks (n=22) | 7428 (6026-8534) |  |

**Table S5: Angiogenesis inhibitor clinical trial results in prostate cancer.**

| **Drug** | **Indication** | **Result** | **Reference** |
| --- | --- | --- | --- |
| Bevacizumab | mCRPC, no prior chemotherapy | Overall survival 22.6 months compared to 21.5 months with placebo (p=0.181); PFS 9.9 months compared to 7.5 months with placebo (p<0.001) | [[12](#_ENREF_12)] |
| Aflibercept | mCRPC, no prior chemotherapy | Overall survival 22.1 months compared to 21.2 months with placebo (p=0.38) | [[13](#_ENREF_13)] |
| Sorafenib | CRPC, no prior chemotherapy | 18/38 patients had ≥ 50% decline in PSA or stable disease ≥ 6 months | [[14](#_ENREF_14)] |
| Sunitinib | mCRPC, docetaxel resistant | Overall survival 13.1 months compared to 11.8 months with placebo (p=0.168); PFS 5.6 months compared to 4.1 months with placebo (p<0.001) | [[15](#_ENREF_15)] |
| Cabozantinib | CRPC, with or without prior chemotherapy | Progression-free survival 23.9 weeks compared to 5.9 weeks with placebo (p<0.001) | [[16](#_ENREF_16)] |

**Table S6: Significant genes in the 478-gene angiome.** Gene names are color-coded based on the class in which the gene is up-regulated. Colors for the first 12 rows correspond to tissue types as in Figure 3C-D of the main text (green = normal, orange = primary, gray = metastasis), while colors for the last 2 rows correspond to aggressive and indolent primary tumors as in Figure 2A-B of the main text (blue = indolent, red = aggressive).

| **Dataset/Comparison** | **LOOCV AUC** | **Top 10 Genes in PLS-DA Classifier** |
| --- | --- | --- |
| TCGA Primary vs. Normal | 0.98 | ***EFNB1, SNCG, CLU, DOK4, ID4, MMP9, COL4A6, FGFR2, IGSF1, THBS2*** |
| GSE21034 Primary vs. Normal | 0.96 | ***DOK4, CLU, ITGA3, EFNA5, MMP14, COL4A6, ITGB3, COL4A2, RUNX1T1, ITGA9*** |
| GSE6919 Primary vs. Normal | 0.67 | ***CCL11, ANPEP, LDLR, FGFR3, AZGP1, CCR2, CCR5, BAI1, VCAN, VEGFA*** |
| GSE35988 Primary vs. Normal | 0.99 | ***SPP1, FGFR2, CD177, LAMB3, COL7A1, VEGFA, EGFR, TP73, TP63, COL18A1*** |
| GSE32269 Metastasis vs. Primary | 1.00 | ***SPP1, SPARC, TNFAIP6, COL1A1, THBS2, CTSK, CD36, MMP9, SERPINE2, FN1*** |
| GSE21034 Metastasis vs. Primary | 0.97 | ***FGF2, ANGPT1, COL4A6, PDGFC, TGFBR3, EPHA3, FGFR2, SERPINF1, ITGA5, TIMP3*** |
| GSE6919 Metastasis vs. Primary | 0.98 | ***EPHB6, TPI1, MATN2, SPP1, VCAN, BCAM, IGFBP6, FYN, FOS, SMG1*** |
| GSE35988 Metastasis vs. Primary | 1.00 | ***DPP4, ANGPT2, ANPEP, SERPINB6, SEMA3C, PLG, ANTXR2, FLT4, TPI1, FYN*** |
| GSE6919 Metastasis vs. Normal | 1.00 | ***EPHB6, FOS, SPP1, MATN2, FYN, FMOD, TPI1, FGFR2, VCAN, IGFBP6*** |
| GSE21034 Metastasis vs. Normal | 1.00 | ***PDGFC, ANGPT1, TIMP3, TGFBR3, MAML2, ITGA5, EPHA3, TP63, SERPINF1, FGFR2*** |
| GSE38241 Metastasis vs. Normal | 1.00 | ***DPP4, FGFR2, ANTXR2, TP63, MAML2, GPC1, PLP1, LAMB3, MYOC, ITGA5*** |
| GSE35988 Metastasis vs. Normal | 1.00 | ***ANPEP, COL4A6, CD177, FGFR2, DPP4, IGSF1, EPHB1, NRG1, TP63, ANTXR2*** |
| TCGA Aggressive vs. Indolent | 0.72 | ***THBS2, VCAN, INHBA, THBS3, ANGPT2, IGFBP3, FN1, COL1A1, ERBB2, AZGP1*** |
| GSE21034 Aggressive vs. Indolent | 0.66 | ***CCL2, IL6, HBEGF, TPI1, NRG1, COL4A6, PDIK1L, AREG, LAMB3, COL4A3*** |

**Table S7: Significant genes in the 1233-gene extended angiome.** Gene names are color-coded based on the class in which the gene is up-regulated. Colors for the first 12 rows correspond to tissue types as in Figure 3C-D of the main text (green = normal, orange = primary, gray = metastasis), while colors for the last 2 rows correspond to aggressive and indolent primary tumors as in Figure 2A-B of the main text (blue = indolent, red = aggressive).

| **Dataset/Comparison** | **LOOCV AUC** | **Top 10 Genes in PLS-DA Classifier** |
| --- | --- | --- |
| TCGA Primary vs. Normal | 0.98 | ***MKI67, RRM2, HMMR, EZH2, FOXM1, BIRC5, PDE3B, SNCG, EFNB1, CLU*** |
| GSE21034 Primary vs. Normal | 0.96 | ***CLU, CAV1, RRAS, LGALS1, DOK4, EFNA5, ITGA3, SPON1, PRKG1, MCAM*** |
| GSE6919 Primary vs. Normal | 0.72 | ***MMP15, CXCR4, MMP25, APOE, CCL3, SDCBP, CD38, CSF1, EPOR, CYBB*** |
| GSE35988 Primary vs. Normal | 0.98 | ***SPP1, APOE, TG, CD177, EGFR, FGFR2, TP73, COL7A1, LAMB3, NRG2*** |
| GSE32269 Metastasis vs. Primary | 1.00 | ***SPP1, COL1A2, SPARC, TNFAIP6, PTTG1, EZH2, ENPEP, THBS2, APOE, COL1A1*** |
| GSE21034 Metastasis vs. Primary | 0.97 | ***CHRDL1, MEIS1, TRPC4, PGR, FGF2, ANGPT1, CRYAB, EDNRA, PDGFC, PTGER2*** |
| GSE6919 Metastasis vs. Primary | 0.99 | ***AR, HSP90AA1, YY1, TUBA1B, BTG1, TARS, TPI1, EPHB6, TFRC, SPP1*** |
| GSE35988 Metastasis vs. Primary | 1.00 | ***LFNG, ANPEP, ANGPT2, SFRP1, DPP4, CCNA2, CD38, SERPINB6, TP63, ANTXR2*** |
| GSE6919 Metastasis vs. Normal | 1.00 | ***TUBA1B, OS9, DUSP1, BTG1, EPHB6, YY1, PTN, FOS, NR4A1, FYN*** |
| GSE21034 Metastasis vs. Normal | 1.00 | ***MEIS1, CHRDL1, PDGFC, TRPC4, CAV1, EDNRA, ANGPT1, VCL, PGR, TIMP3*** |
| GSE38241 Metastasis vs. Normal | 1.00 | ***FGFR2, HOXD10, GATA3, DPP4, LGALS3, CD38, TP63, EDNRA, HSPB1, GPC1*** |
| GSE35988 Metastasis vs. Normal | 1.00 | ***ANPEP, FBLN1, FGFR2, CD177, GRP, SFRP1, CTSG, PGR, TP63, NRG2*** |
| TCGA Aggressive vs. Indolent | 0.74 | ***CD38, THBS3, XBP1, THBS2, MMP3, IGFBP3, OS9, MMP11, MAPK9, FAP*** |
| GSE21034 Aggressive vs. Indolent | 0.69 | ***CDKN1A, HBEGF, EGR1, CCL2, NR4A1, IL6, CX3CL1, NRG1, CRYAB, RHOB*** |

**Figure S1**

**
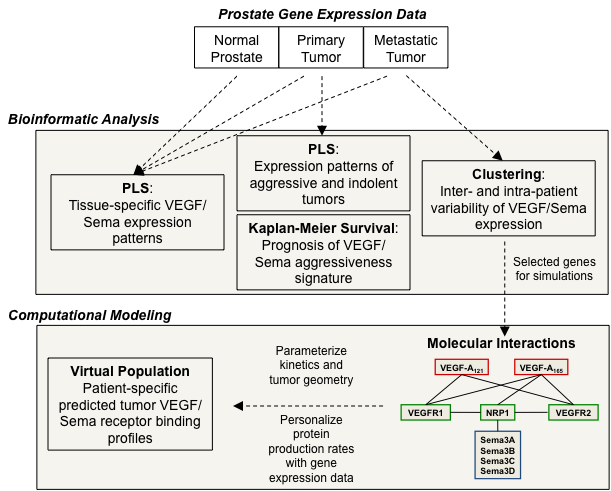
**

**Figure S1: Flowchart of the methods in this study.Figure S2**

**
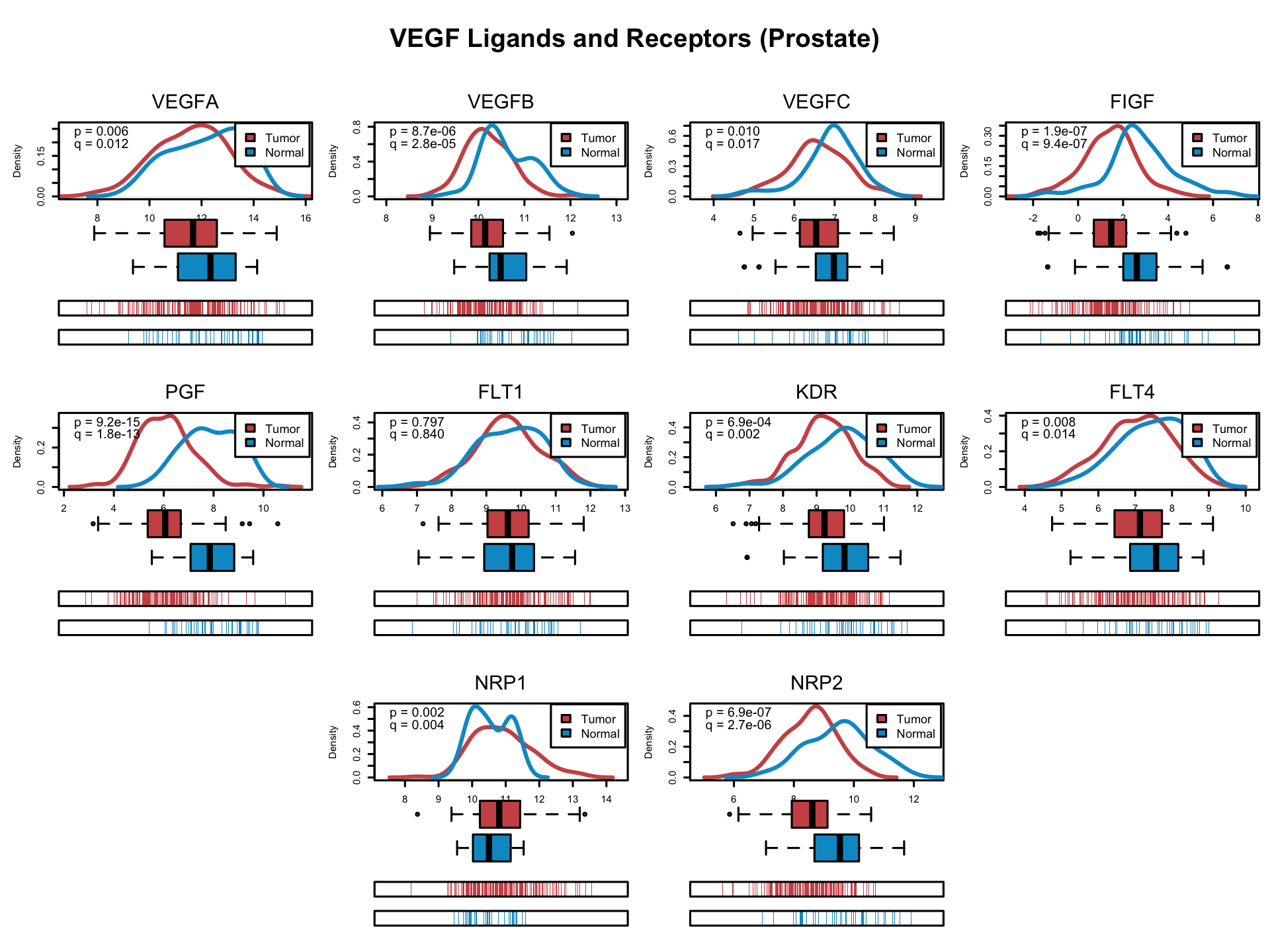
**

**
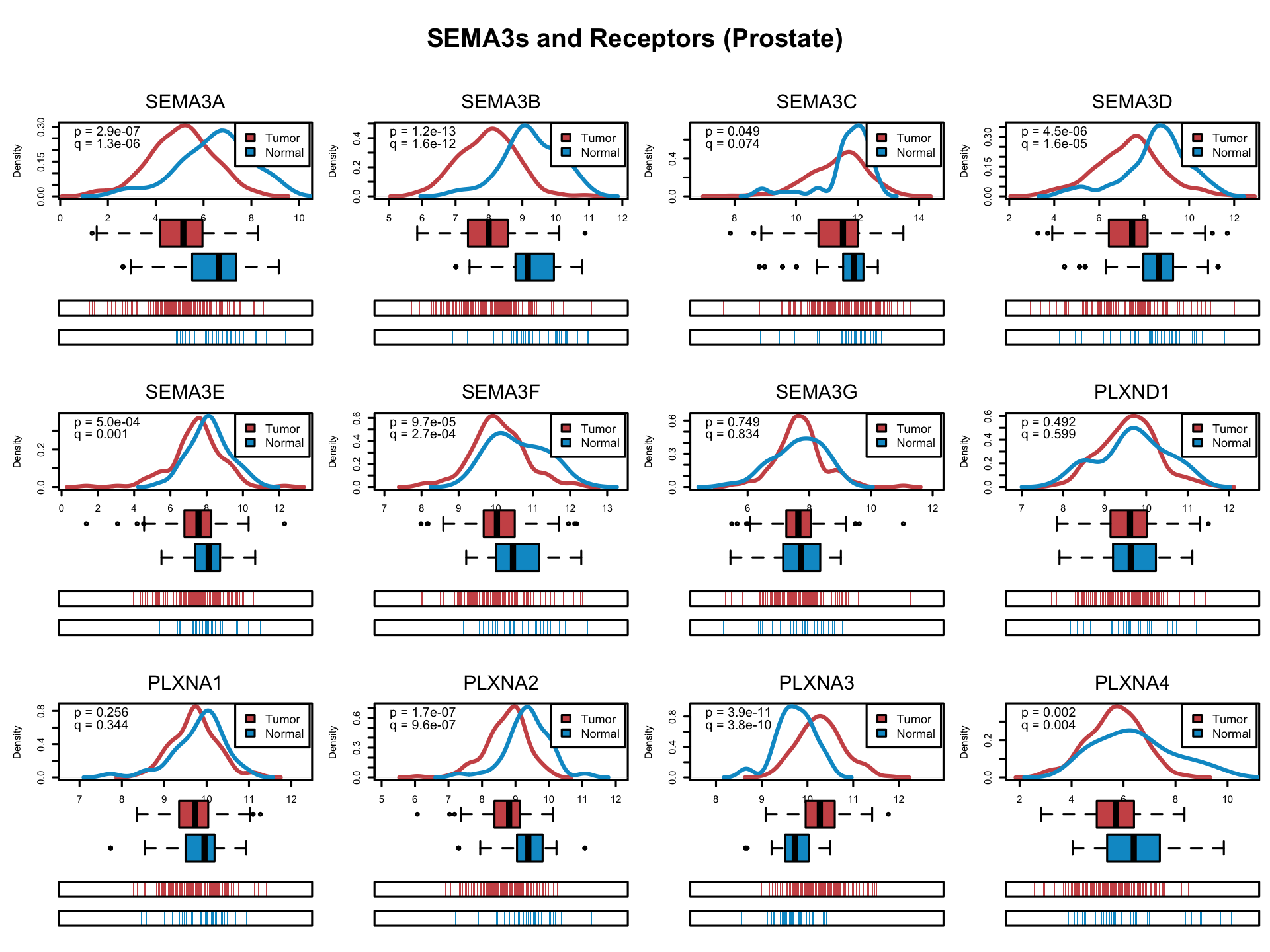
**

**Figure S2: Differential expression of VEGF/Sema3 ligands and receptors in prostate cancer.** This figure expands upon Figure 1A-B from the main text. All data is from the TCGA dataset (n=176 for tumors, n=44 for normal tissues).

**Figure S3**

**
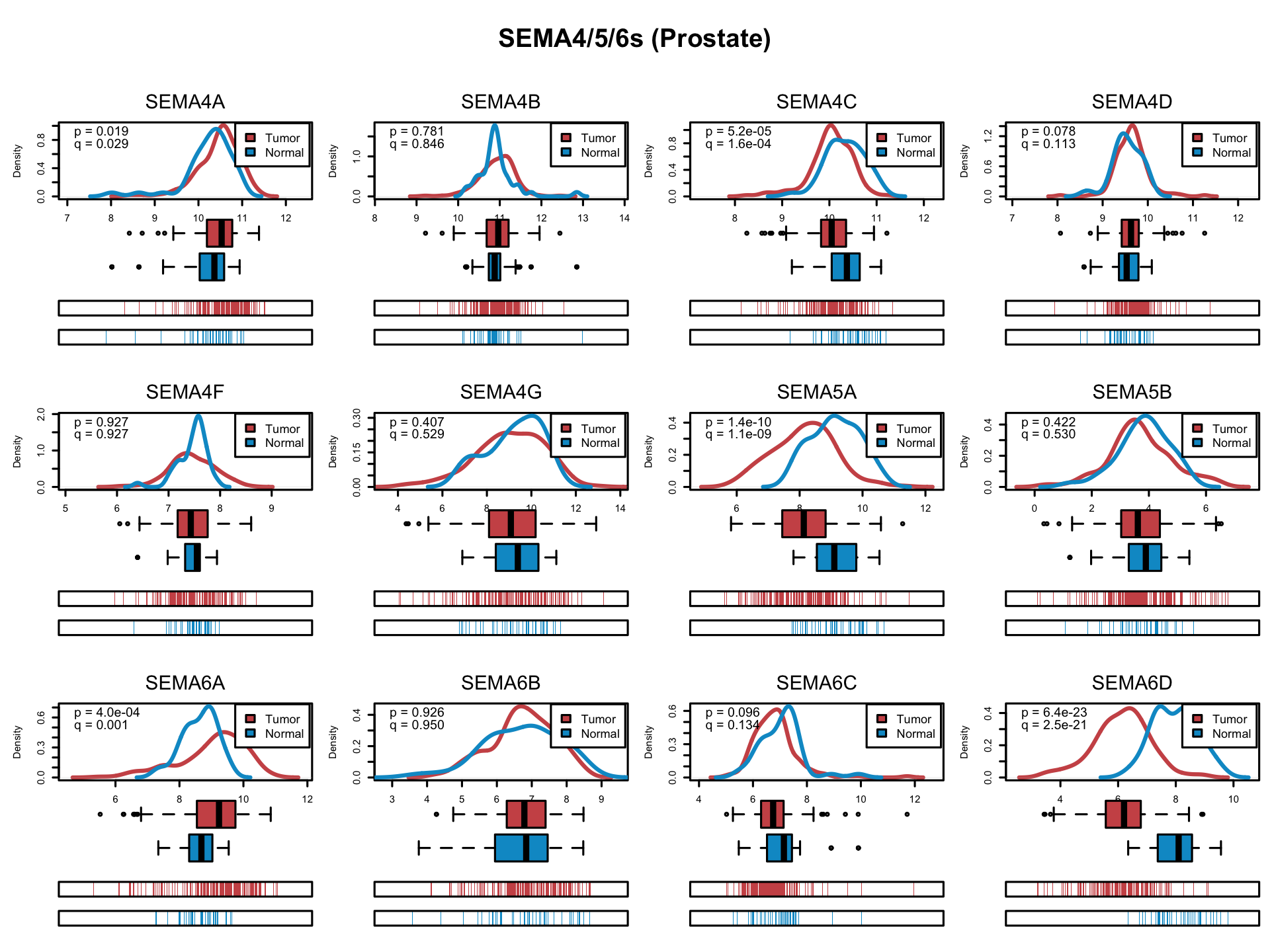
**

**
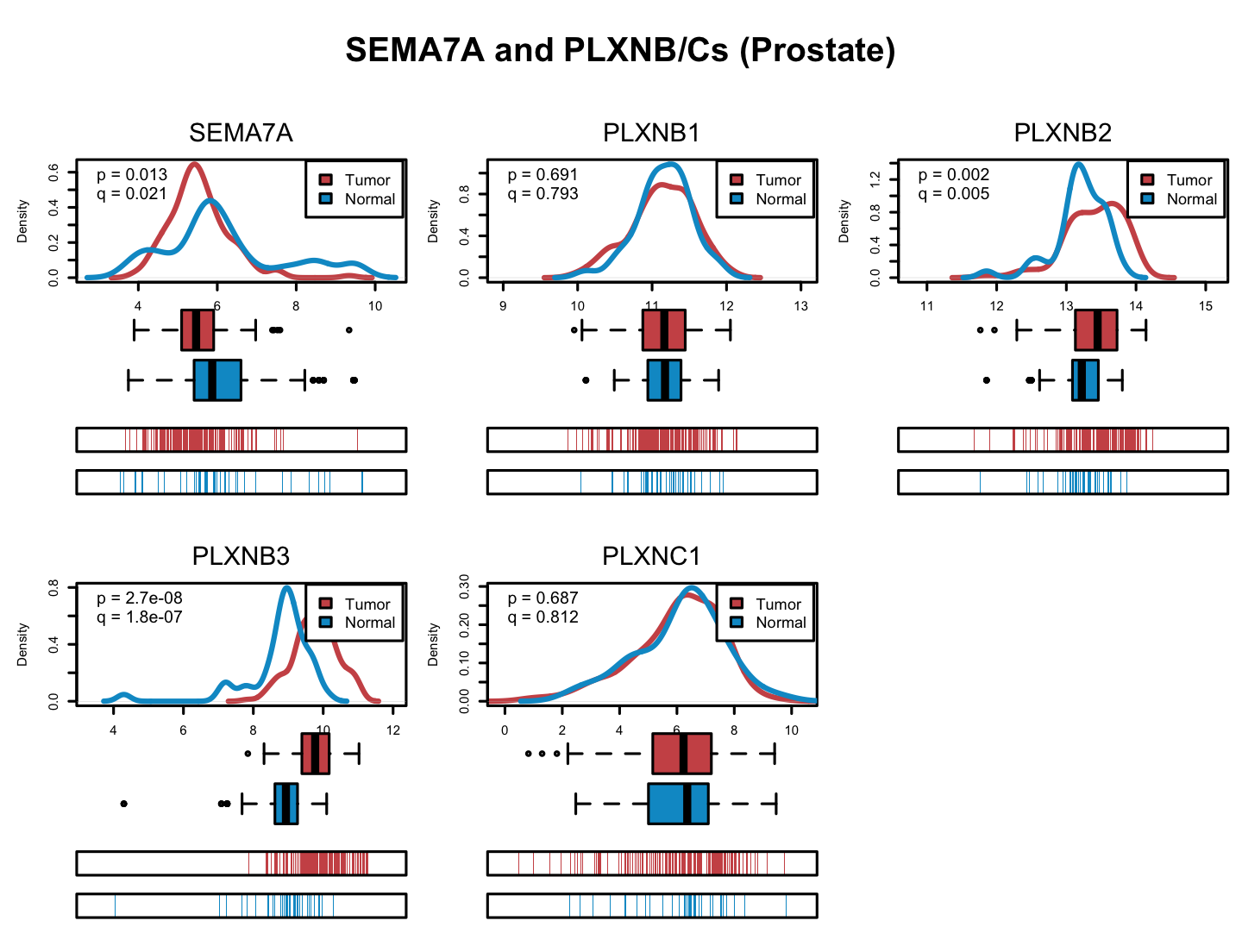
**

**Figure S3: Differential expression of Sema4/5/6/7 ligands and receptors in prostate cancer.** This figure expands upon Figure 1A-B from the main text. All data is from the TCGA dataset (n=176 for tumors, n=44 for normal tissues).

**Figure S4**

**
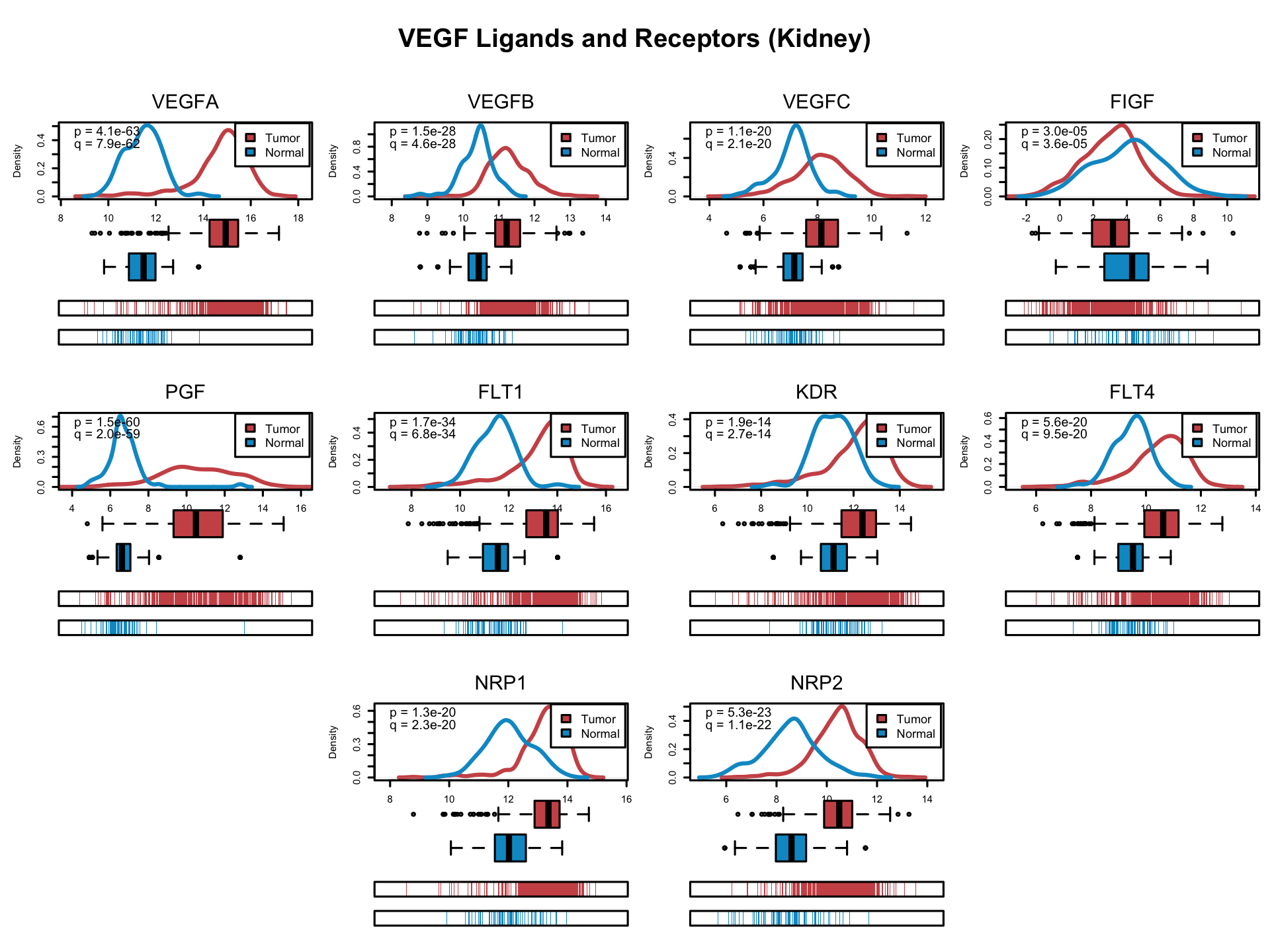
**

**
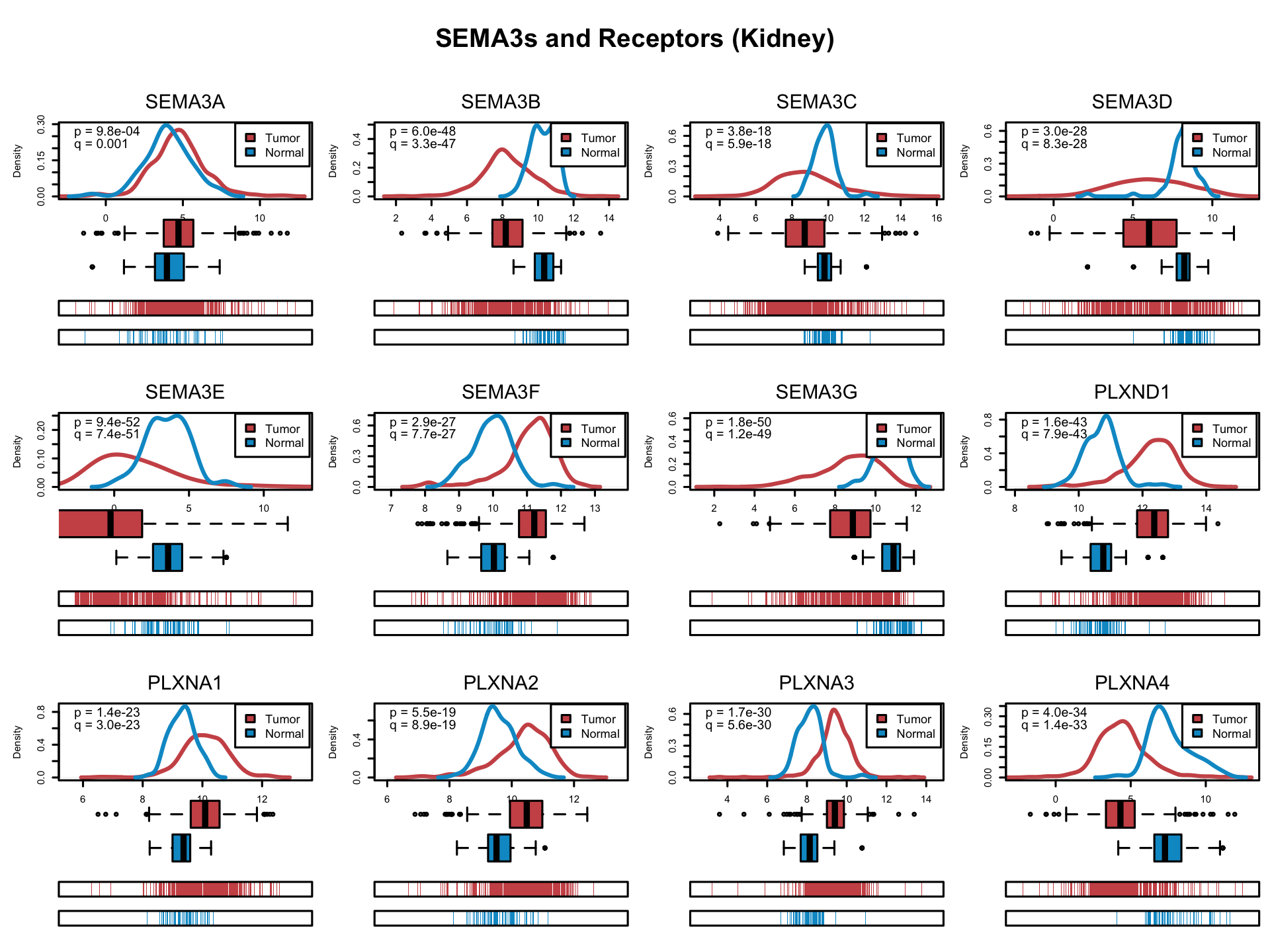
**

**Figure S4: Differential expression of VEGF/Sema3 ligands and receptors in renal cell carcinoma.** This figure expands upon Figure 1C-D from the main text. All data is from the TCGA dataset (n=480 for tumors, n=71 for normal tissues).

**Figure S5**

**
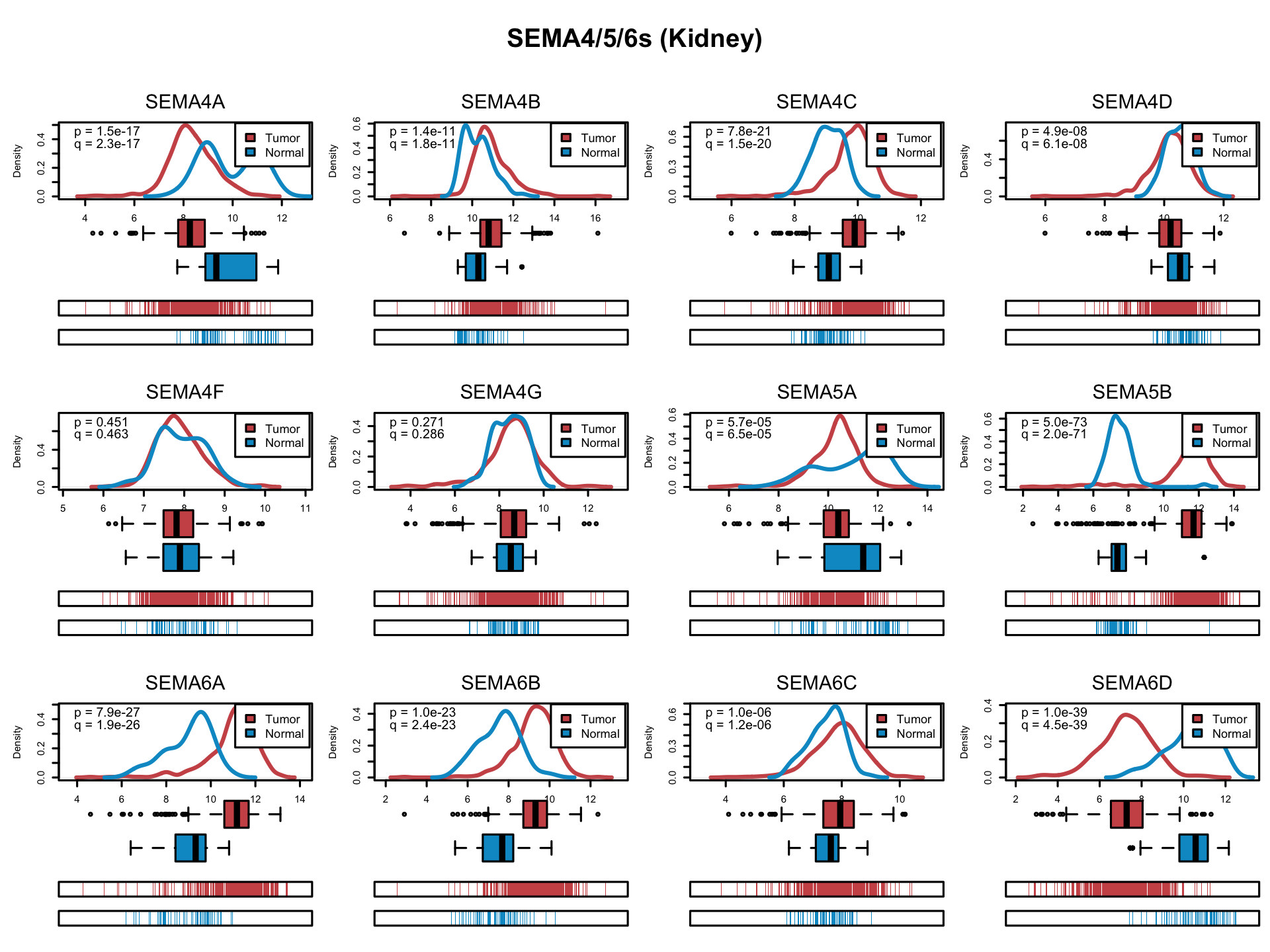
**

**
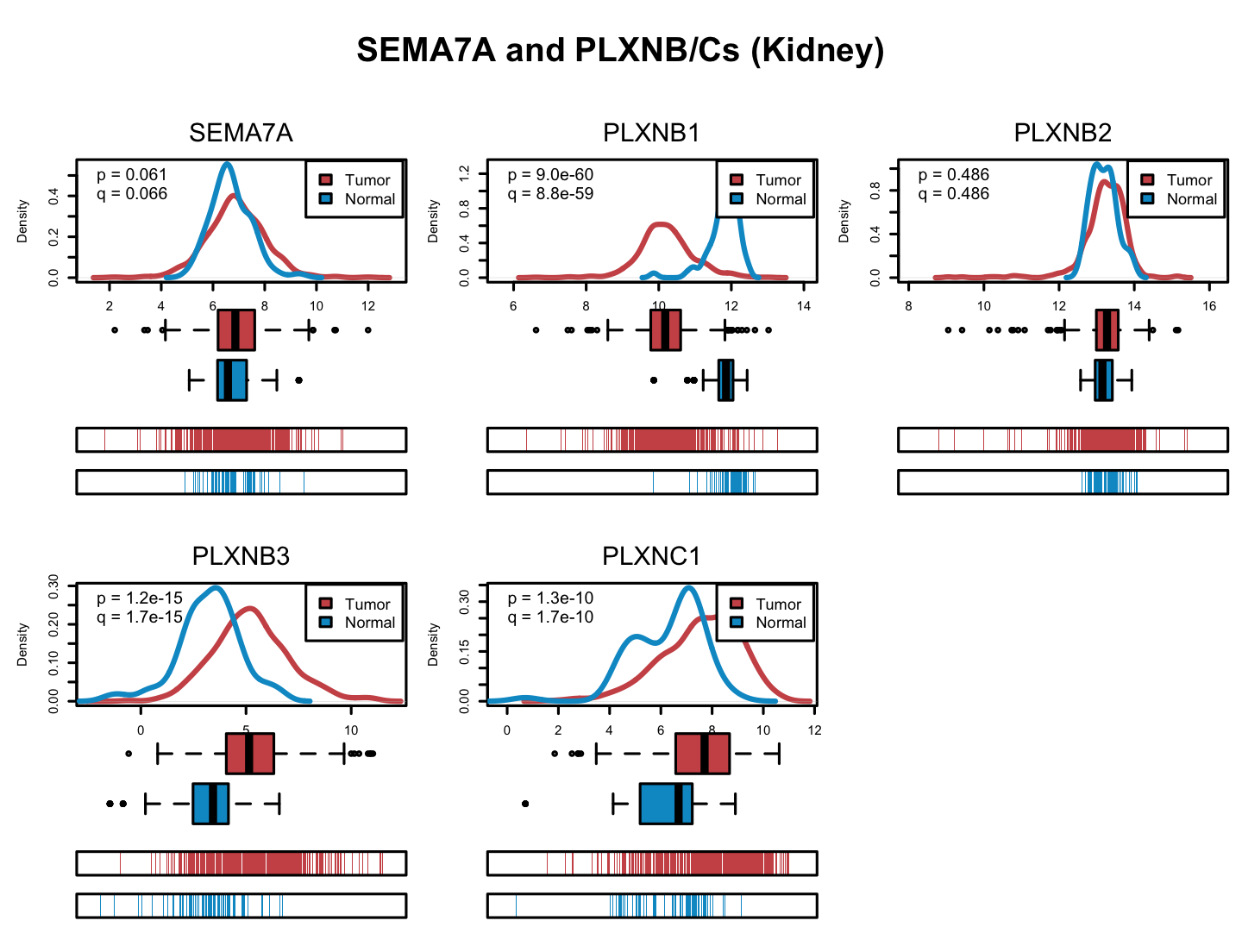
**

**Figure S5: Differential expression of Sema4/5/6/7 ligands and receptors in renal cell carcinoma.** This figure expands upon Figure 1C-D from the main text. All data is from the TCGA dataset (n=480 for tumors, n=71 for normal tissues).**Figure S6**

**
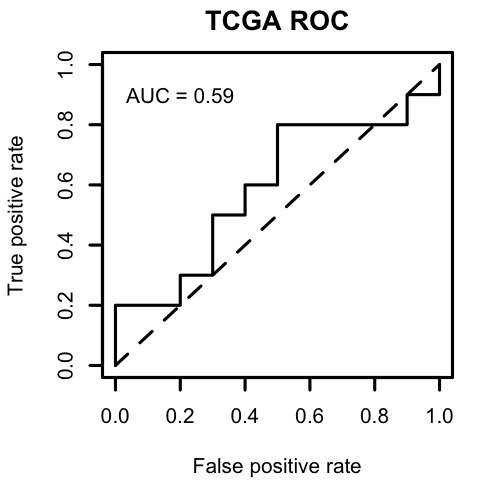

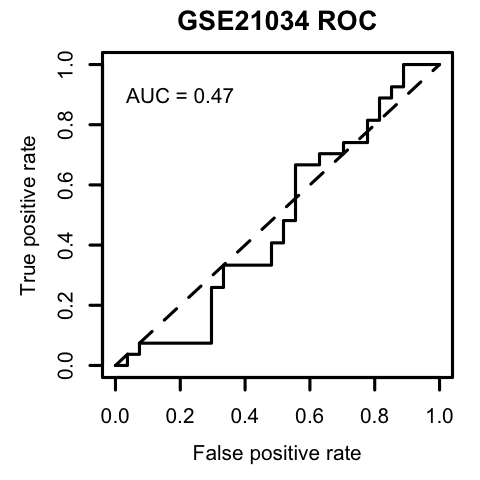
**

**Figure S6: ROC curves for LOOCV of PLS-DA models of VEGF/Sema expression in aggressive and indolent tumors in the TCGA and GSE21034 datasets.** This figure expands upon Figure 2 from the main text.

**Figure S7**


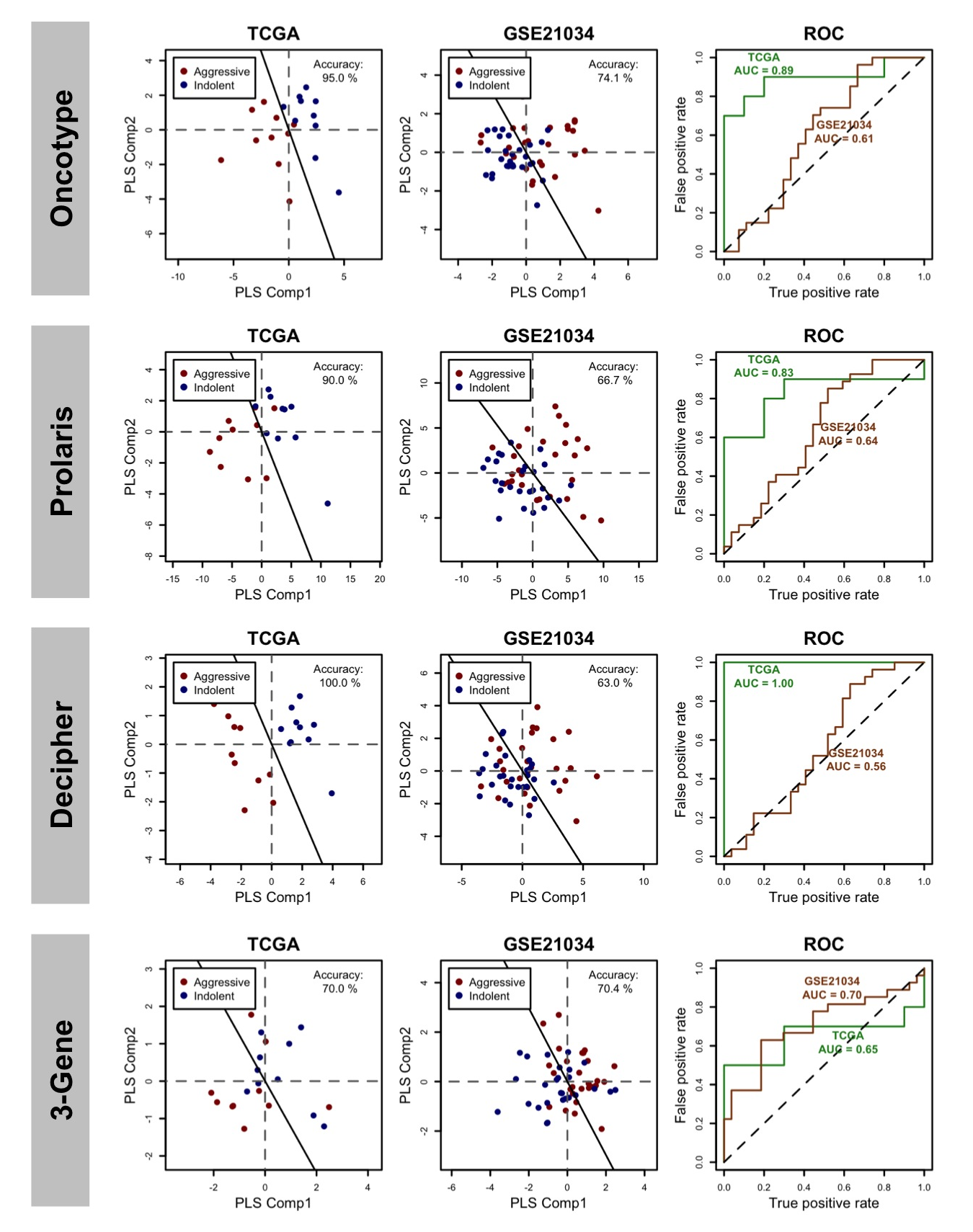


**Figure S7: Biomarker performance in TCGA and GSE21034 datasets.** This figure expands upon Figure 2 from the main text. Classifiers were trained on the aggressive/indolent subset, the same as Figures 2A and 2B in the main text.

**Figure S8**


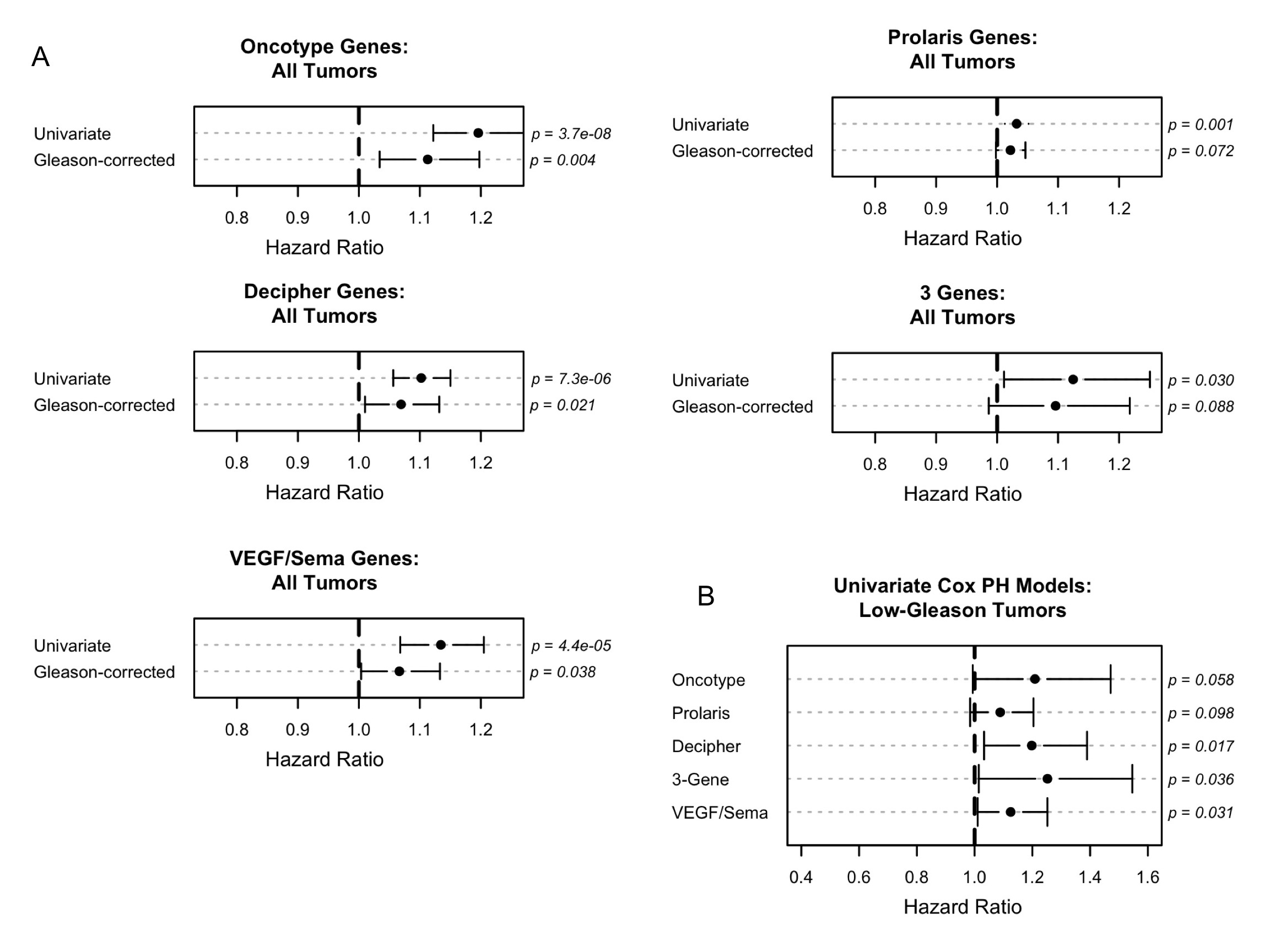


**Figure S8: Cox proportional hazards modeling of the association between PLS-DA biomarkers and biochemical recurrence (BCR).** This figure expands upon Figure 2 from the main text. **A:** Hazard ratios for the four biomarkers in all primary tumors of the GSE21034 dataset (n=131). Both univariate and Gleason score-corrected models are displayed. **B:** Univariate models of primary tumors with Gleason scores equal to 6 or 7 with a primary score of 3 (n=94). The four biomarkers and the VEGF/Sema PLS-DA predictor are shown.

**Figure S9**

**
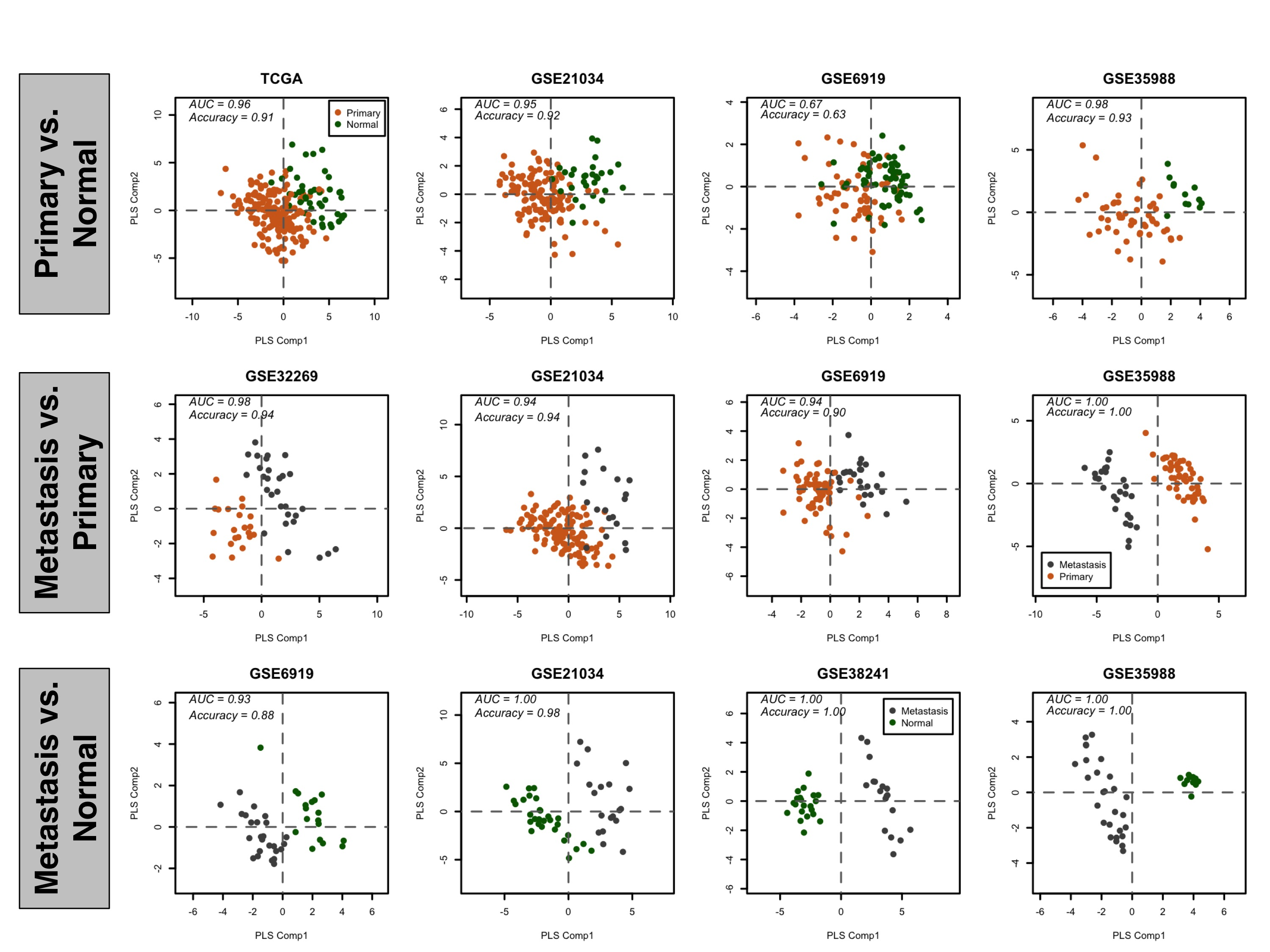
**

**Figure S9: PLS-DA models.** This figure expands upon Figure 3C-D from the main text. PLS-DA models of primary vs. normal, metastasis vs. primary, and metastasis vs. normal in all datasets for which comparisons were possible. The AUC and accuracy values are from leave-one-out cross validation.

**Figure S10**


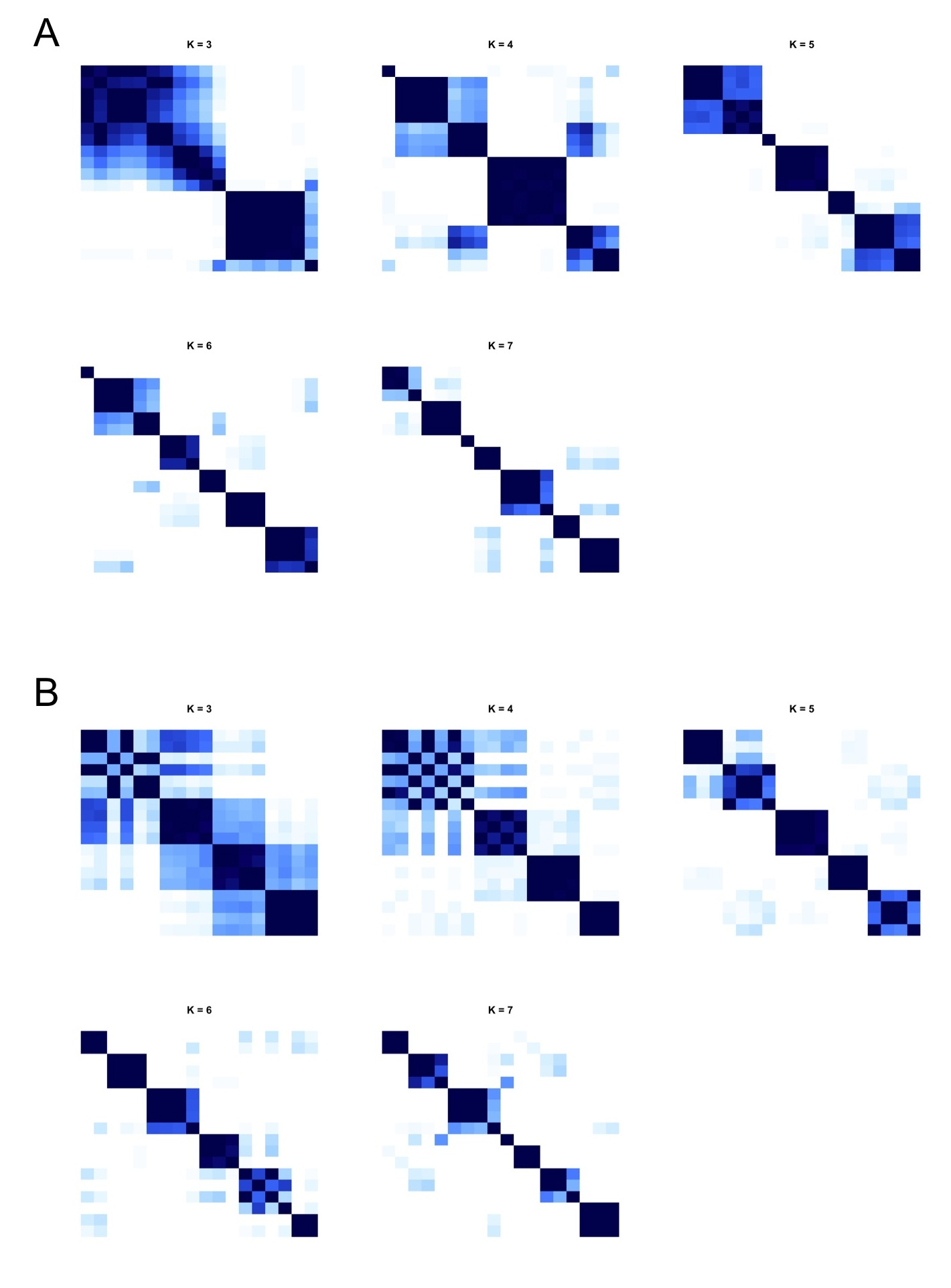


**Figure S10: Consensus *K*-means clustering. A,** consensus plots for K=3-7 for the 39 VEGF/Sema genes in GSE38241. This panel expands upon Figure 4B-C from the main text. **B,** consensus plots for K=3-7 for the 85-gene expanded angiogenic gene set. This panel expands upon Figure 4D-E from the main text.

**Figure S11**

**
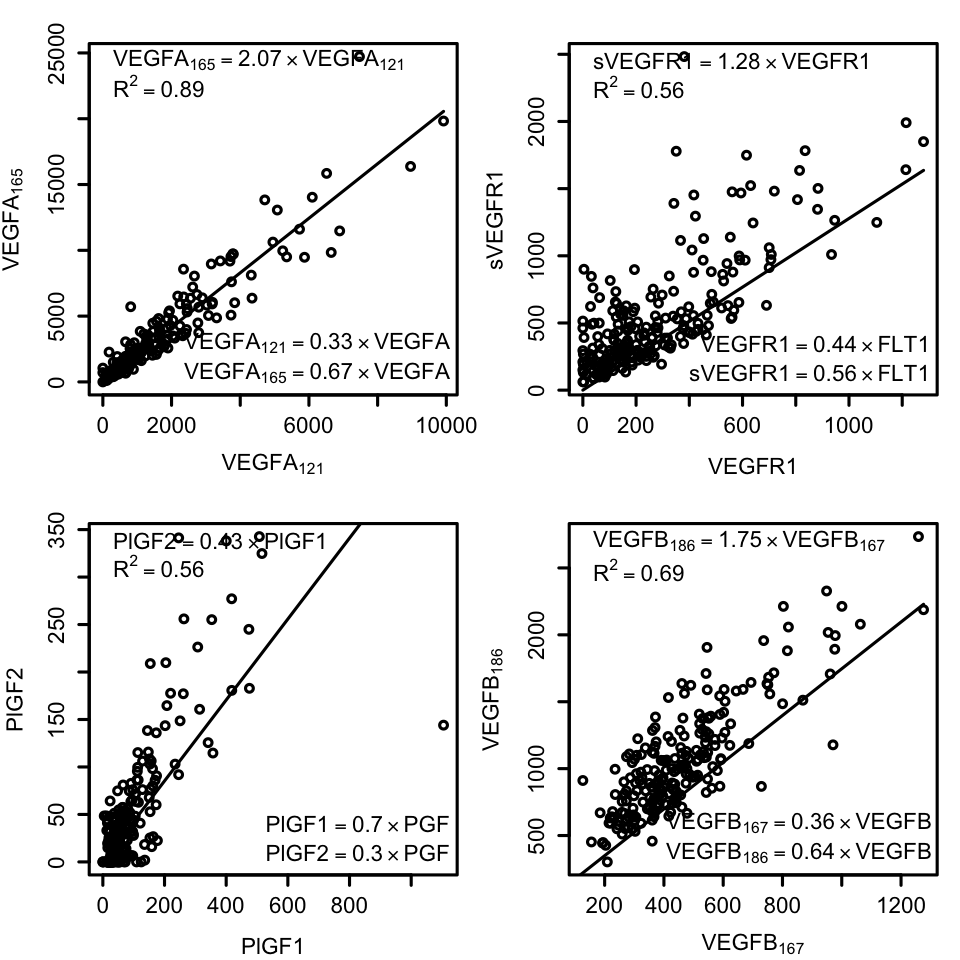
**

**Figure S11: Isoform ratios of genes with alternative splicing.** Four genes known to have multiple isoforms due to alternative transcript splicing were analyzed in the TCGA RNA-Seq dataset. Linear regression models showed a high degree of correlation between pairs of isoforms originating from the same gene. From the linear models, we calculated the relative fraction of total gene expression accounted for by each isoform (bottom right of each plot). These values were used to determine isoform secretion rates in the mathematical model of tumor VEGF/Semaphorin receptor binding.

**Figure S12**


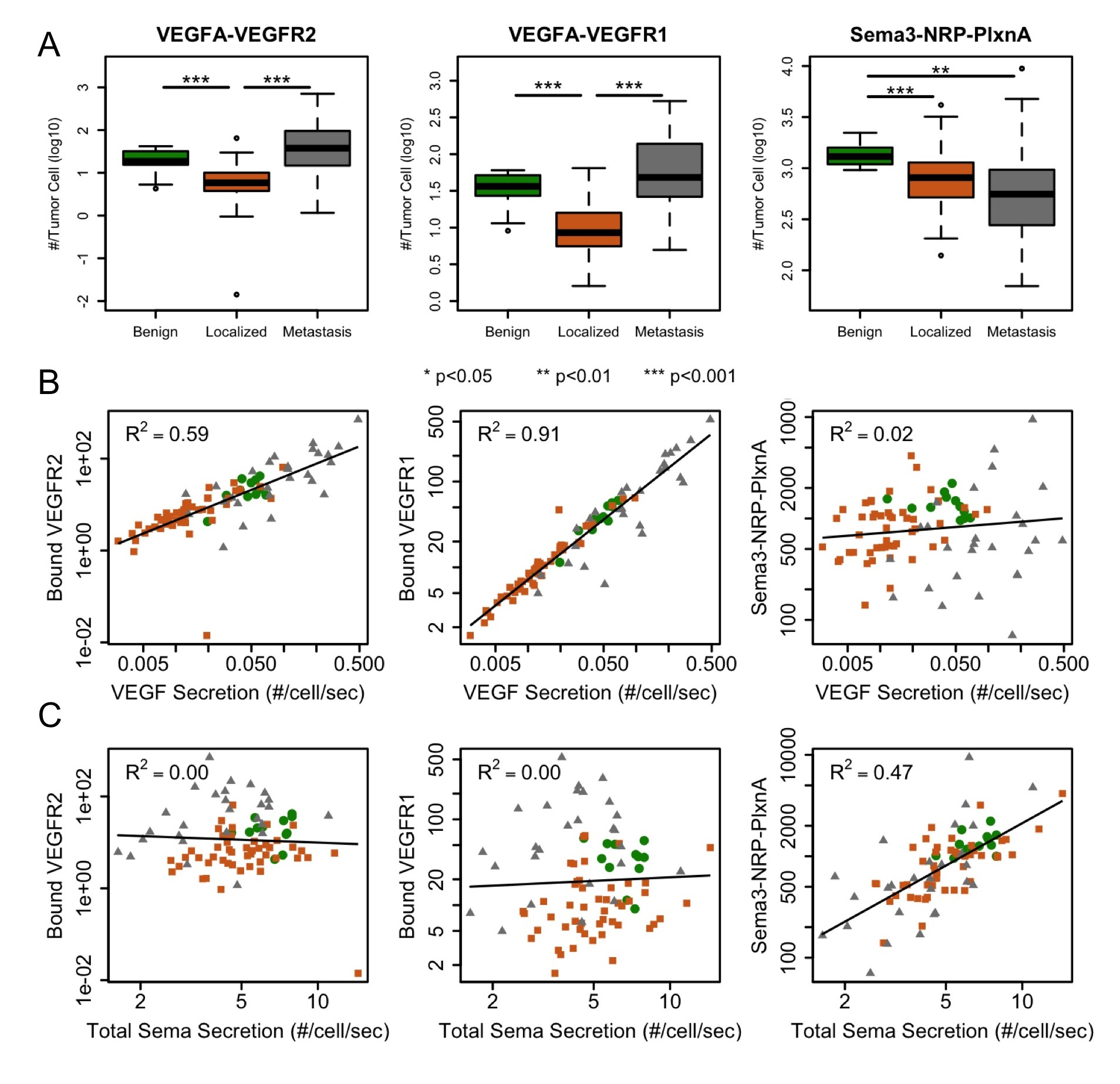


**Figure S12: Tumor cell receptor binding profiles.** This figure expands upon Figure 5 from the main text. Instead of endothelial cells, data are from tumor cells. **A,** Box plots of binding by tissue type (benign, localized, metastasis). **B-C,** Scatter plots showing the effects of VEGF secretion (B) and total Sema3 secretion (C) on the levels of the three complexes listed at the top of the figure.

**SUPPLEMENTAL REFERENCES**

1. Hochberg Y, Benjamini Y: **More powerful procedures for multiple significance testing.** *Stat Med* 1990, **9:**811-818.

2. Taylor BS, Schultz N, Hieronymus H, Gopalan A, Xiao Y, Carver BS, Arora VK, Kaushik P, Cerami E, Reva B, et al: **Integrative genomic profiling of human prostate cancer.** *Cancer Cell* 2010, **18:**11-22.

3. Yu YP, Landsittel D, Jing L, Nelson J, Ren B, Liu L, McDonald C, Thomas R, Dhir R, Finkelstein S, et al: **Gene expression alterations in prostate cancer predicting tumor aggression and preceding development of malignancy.** *J Clin Oncol* 2004, **22:**2790-2799.

4. Cai C, Wang H, He HH, Chen S, He L, Ma F, Mucci L, Wang Q, Fiore C, Sowalsky AG, et al: **ERG induces androgen receptor-mediated regulation of SOX9 in prostate cancer.** *J Clin Invest* 2013, **123:**1109-1122.

5. Aryee MJ, Liu W, Engelmann JC, Nuhn P, Gurel M, Haffner MC, Esopi D, Irizarry RA, Getzenberg RH, Nelson WG, et al: **DNA methylation alterations exhibit intraindividual stability and interindividual heterogeneity in prostate cancer metastases.** *Sci Transl Med* 2013, **5:**169ra110.

6. Grasso CS, Wu YM, Robinson DR, Cao X, Dhanasekaran SM, Khan AP, Quist MJ, Jing X, Lonigro RJ, Brenner JC, et al: **The mutational landscape of lethal castration-resistant prostate cancer.** *Nature* 2012, **487:**239-243.

7. Duque JL, Loughlin KR, Adam RM, Kantoff PW, Zurakowski D, Freeman MR: **Plasma levels of vascular endothelial growth factor are increased in patients with metastatic prostate cancer.** *Urology* 1999, **54:**523-527.

8. Caine GJ, Blann AD, Stonelake PS, Ryan P, Lip GY: **Plasma angiopoietin-1, angiopoietin-2 and Tie-2 in breast and prostate cancer: a comparison with VEGF and Flt-1.** *Eur J Clin Invest* 2003, **33:**883-890.

9. Singh A, Gautam KA, Dalela D, Sankhwar S, Natu S, Sankhwar P, Srivastava A: **Plasma vascular endothelial growth factors A and C in patients undergoing prostatic biopsy and TURP for suspected prostatic neoplasia.** *Asian Pac J Cancer Prev* 2013, **14:**2053-2058.

10. Saylor PJ, Kozak KR, Smith MR, Ancukiewicz MA, Efstathiou JA, Zietman AL, Jain RK, Duda DG: **Changes in biomarkers of inflammation and angiogenesis during androgen deprivation therapy for prostate cancer.** *Oncologist* 2012, **17:**212-219.

11. Fontana A, Galli L, Fioravanti A, Orlandi P, Galli C, Landi L, Bursi S, Allegrini G, Fontana E, Di Marsico R, et al: **Clinical and pharmacodynamic evaluation of metronomic cyclophosphamide, celecoxib, and dexamethasone in advanced hormone-refractory prostate cancer.** *Clin Cancer Res* 2009, **15:**4954-4962.

12. Kelly WK, Halabi S, Carducci M, George D, Mahoney JF, Stadler WM, Morris M, Kantoff P, Monk JP, Kaplan E, et al: **Randomized, double-blind, placebo-controlled phase III trial comparing docetaxel and prednisone with or without bevacizumab in men with metastatic castration-resistant prostate cancer: CALGB 90401.** *J Clin Oncol* 2012, **30:**1534-1540.

13. Tannock IF, Fizazi K, Ivanov S, Karlsson CT, Flechon A, Skoneczna I, Orlandi F, Gravis G, Matveev V, Bavbek S, et al: **Aflibercept versus placebo in combination with docetaxel and prednisone for treatment of men with metastatic castration-resistant prostate cancer (VENICE): a phase 3, double-blind randomised trial.** *Lancet Oncol* 2013, **14:**760-768.

14. Beardsley EK, Hotte SJ, North S, Ellard SL, Winquist E, Kollmannsberger C, Mukherjee SD, Chi KN: **A phase II study of sorafenib in combination with bicalutamide in patients with chemotherapy-naive castration resistant prostate cancer.** *Invest New Drugs* 2012, **30:**1652-1659.

15. Michaelson MD, Oudard S, Ou YC, Sengelov L, Saad F, Houede N, Ostler P, Stenzl A, Daugaard G, Jones R, et al: **Randomized, Placebo-Controlled, Phase III Trial of Sunitinib Plus Prednisone Versus Prednisone Alone in Progressive, Metastatic, Castration-Resistant Prostate Cancer.** *J Clin Oncol* 2014, **32:**76-82.

16. Smith DC, Smith MR, Sweeney C, Elfiky AA, Logothetis C, Corn PG, Vogelzang NJ, Small EJ, Harzstark AL, Gordon MS, et al: **Cabozantinib in patients with advanced prostate cancer: results of a phase II randomized discontinuation trial.** *J Clin Oncol* 2013, **31:**412-419.
