# Supplementary material for: Dysregulation of the vascular endothelial growth factor and semaphorin ligand-receptor families in prostate cancer metastasis
Source: BMC Syst Biol. 2015 Sep 4;9:55. doi: 10.1186/s12918-015-0201-z (PMC4559909; doi:10.1186/s12918-015-0201-z)
Supplement: Additional file 2: — Computational model development, parameters and supplemental figures. Table S8. Geometric parameters. Table S9. Rate constants of ligand-receptor binding reactions. Table S10. Rate constants of receptor coupling reactions. Table S11. Target free ligand concentrations. Table S12. Model parameters. Figure S13. Reactions in the VEGF/Sema model. Figure S14. Receptor fractional occupancies at steady-state. Figure S15. Effect of ligand secretion rates on receptor binding. (DOCX 5884 kb) [file 12918_2015_201_MOESM2_ESM.docx]

***Supplement 2 for***

**Dysregulation of the Vascular Endothelial Growth Factor and Semaphorin ligand-receptor families in prostate cancer metastasis**

R. Joseph Bender and Feilim Mac Gabhann

**Supplement 2: Computational model development, parameters and supplemental figures**

**Supplemental Methods: Computational modeling of VEGF and semaphorin binding 2-4**

**Table S8: Geometric parameters 5**

**Table S9: Rate constants of ligand-receptor binding reactions 6-7**

**Table S10: Rate constants of receptor coupling reactions 8**

**Table S11: Target free ligand concentrations 9**

**Table S12: Model parameters 10**

**Figure S13: Reactions in the VEGF/Sema model. 11-13**

**Figure S14: Receptor fractional occupancies at steady-state. 14**

**Figure S15: Effect of ligand secretion rates on receptor binding. 15**

**Supplemental References 16**

**Supplemental Methods: Computational modeling of VEGF and semaphorin binding**

To assess the effects of patient tumor gene expression on VEGF and semaphorin signaling, we built a model of ligand-receptor binding that encompassed all VEGF proteins for which gene expression data was available, all class 3 semaphorins, and all receptors known to bind these ligands. We expanded our validated kinetic model comprising interactions between two isoforms of VEGF-A (VEGF_121_ and VEGF_165_), VEGFR-1, VEGFR-2, Neuropilin-1, Neuropilin-2 and soluble VEGFR-1 (sVEGFR-1) [[1-4](#_ENREF_1)] to include the additional encoded VEGF and Sema family proteins. The scope of some of these modeling studies has included the entire body so that drug pharmacokinetics could be studied [[2](#_ENREF_2), [3](#_ENREF_3)]; here, we limit the scope of the model to a single compartment representing a tumor and the surrounding microvasculature, justified by our results showing the small size of interaction effects between the tumor compartment and other compartments. We use this model to study only the effects of gene expression variation.

*Geometry.* We assumed that the tumor volume consisted only of tumor cells, endothelial cells, and the interstitial space between these cells. The interstitial space available to ligands and the surface area to volume ratios of the two cell types were as previously described ([[1](#_ENREF_1)] and Table S8).

*Kinetics.* Rate constants for the association and dissociation reactions between the two major VEGF-A isoforms, VEGF-A_165_ and VEGF-A_121_, and VEGFR-1, VEGFR-2, and Neuropilin-1 were as previously described ([[4](#_ENREF_4)], Table S9 and Table S10). The interaction of VEGF-A_165_ with Neuropilin-2 and coupling between Neuropilin-2 and VEGFR-1/VEGFR-2 were as described in [[3](#_ENREF_3)] (Tables S9 and S10). Interactions of sVEGFR-1 with the two VEGF-A isoforms and Neuropilin-1 were as described in [[2](#_ENREF_2)] (Table S9).

For reactions whose rate constants were not directly known, we assumed a k_off_ of 1x10^-3^ sec^-1^ (consistent with measured off rates for this family) and calculated k_on_ as k_off_/K_d_, where K_d_ is the measured dissociation constant obtained from the literature. Dissociation constants for the interactions of PlGF-1 and PlGF-2 with VEGFR-1 were available [[5](#_ENREF_5)], but no data was available for the binding of PlGF-2 to Neuropilin-1, thus it was estimated to be the same as for the binding of VEGF-A_165_ to Neuropilin-1. Dissociation constants were not available for the two isoforms of VEGF-B, VEGF-B_167_ and VEGF-B_186_, for their receptors VEGFR-1 and Neuropilin-1. We used the same rate constants for these two reactions as PlGF-2. Dissociation constants were available for the binding of VEGF-C to VEGFR-2 and VEGFR-3 [[6](#_ENREF_6)], but not for the binding of VEGF-C to Neuropilin-2. In this case, we assumed the same kinetics as the binding of VEGF-A_165_ to Neuropilin-1. No dissociation constants were available for the binding of VEGF-D to its receptors, VEGFR-2, VEGFR-3, and Neuropilin-2, therefore we used the same rate constants as VEGF-C.

For class 3 Semaphorin binding to Neuropilins and Neuropilin/Plexin complexes, dissociation constant data was only available for the binding of Sema3A and Sema3C to Neuropilin-1 and Neuropilin-1/Plexin-A1 complexes, and for the binding of Sema3C and Sema3F to Neuropilin-2 and Neuropilin-2/Plexin-A1 complexes ([[7](#_ENREF_7)] and Table S9). The on rate constants k_on_ for the binding of these Semaphorins to their respective Neuropilins could be estimated directly from the dissociation constants, but the values of k_on_ for binding of Semaphorins to Neuropilin/Plexin complexes had to be determined from simulations due to the presence of both Neuropilin and Neuropilin/Plexin complexes (see supplemental text S2 for details). We assumed that the kinetics of coupling between the Neuropilin and Plexin receptors occurred with identical rates constants to those for VEGFR1 and Neuropilin-1 coupling (Table S10). Data was also available for the binding of Sema3E to its receptor Pleixn-D1 ([[8](#_ENREF_8)] and Table S9).

*Receptor densities.* As the amount of receptors on the surface of cells *in vivo* is difficult to measure, we use quantitative flow cytometry data of cultured cell lines where available. The densities of VEGFR-1, VEGFR-2, and NRP-1 have previously been measured in this way and used in computational models ([[3](#_ENREF_3)] and Table S12). We assumed NRP-2 densities were similar to NRP-1 as in [[3](#_ENREF_3)]. To our knowledge, VEGFR-3 and Plexin levels have not been measured on endothelial or tumor cells; therefore, we set the density of each of these receptors to 1,000, which falls in the middle of the range of cell surface densities in our model.

*Ligand secretion rates.* We adjusted ligand secretion rates so that the steady-state concentrations of free ligands matched pre-determined target concentrations. The target concentrations were derived from published measurements of plasma concentrations in cancer patients. Actual interstitial ligand concentrations would likely be higher than plasma concentrations, but published data of interstitial concentrations are rare due to the difficulty in obtaining these measurements. Data for plasma concentrations of VEGF ligands were widely available due to studies testing their performance as pharmacodynamic markers of VEGF-targeting drugs (Table S11). To our knowledge, only one study has been published with data for plasma Semaphorin concentrations, which found a plasma Sema3A concentration of 74.41 ng/mL in healthy patients [[9](#_ENREF_9)]. Here, we used this order of magnitude (10^4^ pg/mL) for each of the seven class 3 Semaphorins (Table S11).

*Variation due to gene expression.* Gene expression data was used to vary the protein production rates in tumor cells only. The log2-transformed gene expression data was first median-centered and then added to the log2-transformed nominal production rates found in the previous section. The actual production rate was then found by exponentiation with base 2. Thus the median gene expression corresponds to the nominal secretion rate found in the previous section. For *VEGFA*, *PGF*, *VEGFB*, and *FLT1*, two isoforms were represented in the model for each gene. We used TCGA data to determine average isoform fractions of total gene expression (Supplementary Figure S11 in File S1). Isoforms from identical genes were typically highly correlated.

The system of coupled nonlinear differential equations describing the amounts of each molecule/complex was solved numerically in Fortran using the fifth-order Runge-Kutta method with adaptive step-size control.

**Table S8: Geometric parameters**

| **Parameter** | **Value** | **Units** | **Reference** |
| --- | --- | --- | --- |
| Microvessel surface area to volume ratio | 105 | cm^2^/cm^3^ tissue | [[1](#_ENREF_1)] |
| Tumor surface area to volume ratio | 1534 | cm^2^/cm^3^ tissue | [[1](#_ENREF_1)] |
| Endothelial cell surface area | 1•10^-5^ | cm^2^ | [[1](#_ENREF_1)] |
| Tumor cell surface area | 1•10^-5^ | cm^2^ | [[1](#_ENREF_1)] |
| Interstitial volume fraction | 0.58 | cm^3^/cm^3^ tissue | [[1](#_ENREF_1)] |

**Table S9: Rate constants of ligand-receptor binding reactions**

| **Reaction** | **k_on_**  **(M^-1^sec^-1^)** | **k_off_**  **(sec^-1^)** | **K_d_**  **(pM)** | **Ref** |
| --- | --- | --- | --- | --- |
| VEGFA_165_ + VEGFR1 ↔ VEGFA_165_-R1 | 3•10^7^ | 1•10^-3^ | 33 | [[1](#_ENREF_1)] |
| VEGFA_165_ + VEGFR2 ↔ VEGFA_165_-R2 | 1•10^7^ | 1•10^-3^ | 100 | [[1](#_ENREF_1)] |
| VEGFA_165_ + NRP1 ↔ VEGFA_165_-NRP1 | 3.125•10^6^ | 1•10^-3^ | 320 | [[1](#_ENREF_1)] |
| VEGFA_165_ + NRP2 ↔ VEGFA_165_-NRP2 | 1•10^6^ | 1•10^-3^ | 320 | [[1](#_ENREF_1)] |
| VEGFA_121_ + VEGFR1 ↔ VEGFA_121_-R1 | 3•10^7^ | 1•10^-3^ | 33 | [[1](#_ENREF_1)] |
| VEGFA_121_ + VEGFR2 ↔ VEGFA_121_-R2 | 1•10^7^ | 1•10^-3^ | 100 | [[1](#_ENREF_1)] |
| VEGFA_121_ + R1-NRP1 ↔ VEGFA_121_-R1-NRP1 | 3•10^7^ | 1•10^-3^ | 33 | [[1](#_ENREF_1)] |
| VEGFA_121_ + R1-NRP2 ↔ VEGFA_121_-R1-NRP2 | 3•10^7^ | 1•10^-3^ | 33 | [[1](#_ENREF_1)] |
| PlGF1 + VEGFR1 ↔ PlGF1-R1 | 4.3•10^6^ | 1•10^-3^ | 230 | [[5](#_ENREF_5)] |
| PlGF1 + R1-NRP1 ↔ PlGF1-R1-NRP1 | 4.3•10^6^ | 1•10^-3^ | 230 | ^b^ |
| PlGF1 + R1-NRP2 ↔ PlGF1-R1-NRP2 | 4.3•10^6^ | 1•10^-3^ | 230 | ^b^ |
| PlGF2 + VEGFR1 ↔ PlGF2-R1 | 4.3•10^6^ | 1•10^-3^ | 230 | [[5](#_ENREF_5)] |
| PlGF2 + NRP1 ↔ PlGF2-NRP1 | 3.125•10^6^ | 1•10^-3^ | 320 | ^a^ |
| PlGF2 + NRP2 ↔ PlGF2-NRP2 | 3.125•10^6^ | 1•10^-3^ | 320 | ^a^ |
| VEGFB_167_ + VEGFR1 ↔ VEGFB_167_-R1 | 4.3•10^6^ | 1•10^-3^ | 230 | ^b^ |
| VEGFB_186_ + VEGFR1 ↔ VEGFB_186_-R1 | 4.3•10^6^ | 1•10^-3^ | 230 | ^b^ |
| VEGFB_167_ + NRP1 ↔ VEGFB_167_-NRP1 | 3.125•10^6^ | 1•10^-3^ | 320 | ^a^ |
| VEGFB_186_ + NRP1 ↔ VEGFB_186_-NRP1 | 3.125•10^6^ | 1•10^-3^ | 320 | ^a^ |
| VEGFC + VEGFR2 ↔ VEGFC-R2 | 2.4•10^6^ | 1•10^-3^ | 410 | [[6](#_ENREF_6)] |
| VEGFC + VEGFR3 ↔ VEGFC-R3 | 7.4•10^6^ | 1•10^-3^ | 135 | [[6](#_ENREF_6)] |
| VEGFC + NRP2 ↔ VEGFC-NRP2 | 3.125•10^6^ | 1•10^-3^ | 320 | ^a^ |
| VEGFD + VEGFR2 ↔ VEGFD-R2 | 2.4•10^6^ | 1•10^-3^ | 410 | ^c^ |
| VEGFD + VEGFR3 ↔ VEGFD-R3 | 7.4•10^6^ | 1•10^-3^ | 135 | ^c^ |
| VEGFD + NRP2 ↔ VEGFD-NRP2 | 3.125•10^6^ | 1•10^-3^ | 320 | ^a^ |
| Sema3A + NRP1 ↔ Sema3A-NRP1 | 1•10^6^ | 1•10^-3^ | 1000 | [[7](#_ENREF_7)] |
| Sema3B + NRP1 ↔ Sema3B-NRP1 | 1•10^6^ | 1•10^-3^ | 1000 | ^d^ |
| Sema3B + NRP2 ↔ Sema3B-NRP2 | 1•10^6^ | 1•10^-3^ | 1000 | ^d^ |
| Sema3C + NRP1 ↔ Sema3C-NRP1 | 7.7•10^5^ | 1•10^-3^ | 1300 | [[7](#_ENREF_7)] |
| Sema3C + NRP2 ↔ Sema3C-NRP2 | 5.9•10^5^ | 1•10^-3^ | 1700 | [[7](#_ENREF_7)] |
| Sema3D + NRP1 ↔ Sema3D-NRP1 | 1•10^6^ | 1•10^-3^ | 1000 | ^d^ |
| Sema3D + NRP2 ↔ Sema3D-NRP2 | 1•10^6^ | 1•10^-3^ | 1000 | ^d^ |
| Sema3E + PLXND1 ↔ Sema3E-PD1 | 7.7•10^6^ | 1•10^-3^ | 130 | [[8](#_ENREF_8)] |
| Sema3F + NRP2 ↔ Sema3F-NRP2 | 6.7•10^5^ | 1•10^-3^ | 1500 | [[7](#_ENREF_7)] |
| Sema3G + NRP2 ↔ Sema3G-NRP2 | 1•10^6^ | 1•10^-3^ | 1000 | ^d^ |
| Sema3A + PlxnAi-NRP1 ↔ Sema3A-NRP1-PAi | 6.3•10^6^ | 1•10^-3^ | 190 | [[7](#_ENREF_7)]^e^ |
| Sema3A + PlxnD1-NRP1 ↔ Sema3A-NRP1-PD1 | 1•10^6^ | 1•10^-3^ | 1000 | ^d^ |
| Sema3B + PlxnAi-NRP1 ↔ Sema3B-NRP1-PAi | 1•10^6^ | 1•10^-3^ | 1000 | ^d^ |
| Sema3B + PlxnAi-NRP2 ↔ Sema3B-NRP2-PAi | 1•10^6^ | 1•10^-3^ | 1000 | ^d^ |
| Sema3C + PlxnAi-NRP1 ↔ Sema3C-NRP1-PAi | 8.5•10^5^ | 1•10^-3^ | 1200 | [[7](#_ENREF_7)] ^e^ |
| Sema3C + PlxnAi-NRP2 ↔ Sema3C-NRP2-PAi | 3.9•10^5^ | 1•10^-3^ | 2300 | [[7](#_ENREF_7)] ^e^ |
| Sema3C + PlxnD1-NRP1 ↔ Sema3C-NRP1-PD1 | 1•10^6^ | 1•10^-3^ | 1000 | ^d^ |
| Sema3C + PlxnD1-NRP2 ↔ Sema3C-NRP2-PD1 | 1•10^6^ | 1•10^-3^ | 1000 | ^d^ |
| Sema3D + PlxnAi-NRP1 ↔ Sema3D-NRP1-PAi | 1•10^6^ | 1•10^-3^ | 1000 | ^d^ |
| Sema3D + PlxnAi-NRP2 ↔ Sema3D-NRP2-PAi | 1•10^6^ | 1•10^-3^ | 1000 | ^d^ |
| Sema3F + PlxnAi-NRP2 ↔ Sema3F-NRP2-PAi | 2.7•10^6^ | 1•10^-3^ | 440 | [[7](#_ENREF_7)] ^e^ |
| Sema3G + PlxnAi-NRP2 ↔ Sema3G-NRP2-PAi | 1•10^6^ | 1•10^-3^ | 1000 | ^d^ |
| VEGF_165_ + sVEGFR1 ↔ VEGF_165_-sR1 | 3•10^7^ | 1•10^-3^ | 33 | [[2](#_ENREF_2)] |
| VEGF_121_ + sVEGFR1 ↔ VEGF_121_-sR1 | 3•10^7^ | 1•10^-3^ | 33 | [[2](#_ENREF_2)] |
| PlGF1 + sVEGFR1 ↔ PlGF1-sR1 | 4.3•10^6^ | 1•10^-3^ | 230 | ^b^ |
| PlGF2 + sVEGFR1 ↔ PlGF2-sR1 | 4.3•10^6^ | 1•10^-3^ | 230 | ^b^ |
| VEGFB_167_ + sVEGFR1 ↔ VEGFB_167_-sR1 | 4.3•10^6^ | 1•10^-3^ | 230 | ^b^ |
| VEGFB_186_ + sVEGFR1 ↔ VEGFB_186_-sR1 | 4.3•10^6^ | 1•10^-3^ | 230 | ^b^ |
| sVEGFR1 + NRP1 ↔ sR1-NRP1 | 5.56•10^6^ | 1•10^-2^ | 1800 | [[2](#_ENREF_2)] |
| sVEGFR1 + NRP2 ↔ sR1-NRP2 | 5.56•10^6^ | 1•10^-2^ | 1800 | ^f^ |
| VEGF_121_ + sR1-NRP1 ↔ VEGF_121_-sR1-NRP1 | 3•10^7^ | 1•10^-3^ | 33 | [[2](#_ENREF_2)] |
| PlGF1 + sR1-NRP1 ↔ PlGF1-sR1-NRP1 | 4.3•10^6^ | 1•10^-3^ | 230 | ^b^ |
| VEGF_121_ + sR1-NRP2 ↔ VEGF_121_-sR1-NRP2 | 3•10^7^ | 1•10^-3^ | 33 | [[2](#_ENREF_2)] |
| PlGF1 + sR1-NRP2 ↔ PlGF1-sR1-NRP2 | 4.3•10^6^ | 1•10^-3^ | 230 | ^b^ |
| VEGF_121_-sR1 + NRP1 ↔ VEGF_121_-sR1-NRP1 | 5.56•10^6^ | 1•10^-2^ | 1800 | [[2](#_ENREF_2)] |
| PlGF1-sR1 + NRP1 ↔ PlGF1-sR1-NRP1 | 5.56•10^6^ | 1•10^-2^ | 1800 | ^f^ |
| VEGF_121_-sR1 + NRP2 ↔ VEGF_121_-sR1-NRP2 | 5.56•10^6^ | 1•10^-2^ | 1800 | ^f^ |
| PlGF1-sR1 + NRP2 ↔ PlGF1-sR1-NRP2 | 5.56•10^6^ | 1•10^-2^ | 1800 | ^f^ |
| VEGF_165_ + ECM ↔ VEGF_165_-ECM | 4.2•10^5^ | 1•10^-2^ | 23800 | [[1](#_ENREF_1)] |
| PlGF2 + ECM ↔ PlGF2-ECM | 1•10^7^ | 1•10^-2^ | 1000 | [[10](#_ENREF_10)]^g^ |
| sVEGFR1 + ECM ↔ sR1-ECM | 4.2•10^5^ | 1•10^-2^ | 23800 | [[2](#_ENREF_2)] |
| ^a^ No K_d_ data available; assumed same as K_d_ for VEGFA_165_-NRP1  ^b^ No K_d_ data available; assumed same as K_d_ for PlGF-VEGFR1  ^c^ No K_d_ data available; assumed same as K_d_s for VEGFC binding to VEGFR-2 and VEGFR-3  ^d^ No K_d_ data available; assumed K_d_ of 1 nM (the middle of the range of available Sema3 K_d_s)  ^e^ The K_d_ listed is an overall K_d_ for the cells expressing both NRP1 and PlxnA1  ^f^ Assumed to be the same as sVEGFR1 binding to NRP1  ^g^ In [[10](#_ENREF_10)], PlGF2 on average has 26-fold lower affinity for ECM proteins that VEGFA_165_ | | | | |

**Table S10: Rate constants of receptor coupling reactions**

| **Reaction** | **k_c_**  **((mol/cm^2^)^-1^sec^-1^)** | **k_dis_**  **(sec^-1^)** | **Reference** |
| --- | --- | --- | --- |
| VEGFR1 + NRP1 ↔ R1-NRP1 | 1•10^14^ | 1•10^-2^ | [[1](#_ENREF_1)] |
| VEGFR1 + NRP2 ↔ R1-NRP2 | 1•10^14^ | 1•10^-2^ | [[3](#_ENREF_3)] |
| PlxnAi + NRPj ↔ PAi-NRPj | 1•10^14^ | 1•10^-2^ | ^a^ |
| PlxnD1 + NRPj ↔ PD1-NRPj | 1•10^14^ | 1•10^-2^ | ^a^ |
| VEGF_121_-R1 + NRP1 ↔ VEGF_121_-R1-NRP1 | 1•10^14^ | 1•10^-2^ | [[1](#_ENREF_1)] |
| PlGF1-R1 + NRP1 ↔ PlGF1-R1-NRP1 | 1•10^14^ | 1•10^-2^ | [[1](#_ENREF_1)] |
| VEGF_165_-R2 + NRP1 ↔ VEGF_165_-R2-NRP1 | 3.1•10^13^ | 1•10^-3^ | [[1](#_ENREF_1)] |
| VEGF_165_-NRP1 + VEGFR2 ↔ VEGF_165_-R2-NRP1 | 1•10^14^ | 1•10^-3^ | [[1](#_ENREF_1)] |
| VEGF_165_-R2 + NRP2 ↔ VEGF_165_-R2-NRP2 | 3.1•10^13^ | 1•10^-3^ | [[3](#_ENREF_3)] |
| VEGF_165_-NRP2 + VEGFR2 ↔ VEGF_165_-R2-NRP2 | 1•10^14^ | 1•10^-3^ | [[3](#_ENREF_3)] |
| VEGFC-R2 + NRP2 ↔ VEGFC-R2-NRP2 | 3.1•10^13^ | 1•10^-3^ | [[3](#_ENREF_3)] |
| VEGFC-NRP2 + VEGFR2 ↔ VEGFC-R2-NRP2 | 1•10^14^ | 1•10^-3^ | [[3](#_ENREF_3)] |
| VEGFC-R3 + NRP2 ↔ VEGFC-R3-NRP2 | 3.1•10^13^ | 1•10^-3^ | [[3](#_ENREF_3)] |
| VEGFC-NRP2 + VEGFR3 ↔ VEGFC-R3-NRP2 | 1•10^14^ | 1•10^-3^ | [[3](#_ENREF_3)] |
| VEGFD-R2 + NRP2 ↔ VEGFD-R2-NRP2 | 3.1•10^13^ | 1•10^-3^ | [[3](#_ENREF_3)] |
| VEGFD-NRP2 + VEGFR2 ↔ VEGFD-R2-NRP2 | 1•10^14^ | 1•10^-3^ | [[3](#_ENREF_3)] |
| VEGFD-R3 + NRP2 ↔ VEGFD-R3-NRP2 | 3.1•10^13^ | 1•10^-3^ | [[3](#_ENREF_3)] |
| VEGFD-NRP2 + VEGFR3 ↔ VEGFD-R3-NRP2 | 1•10^14^ | 1•10^-3^ | [[3](#_ENREF_3)] |
| Sema3A-NRP1 + PlxnAi ↔ Sema3A-NRP1-PAi | 1•10^14^ | 1•10^-2^ | ^a^ |
| Sema3A-NRP1 + PlxnD1 ↔ Sema3A-NRP1-PD1 | 1•10^14^ | 1•10^-2^ | ^a^ |
| Sema3B-NRP1 + PlxnAi ↔ Sema3B-NRP1-PAi | 1•10^14^ | 1•10^-2^ | ^a^ |
| Sema3B-NRP2 + PlxnAi ↔ Sema3B-NRP2-PAi | 1•10^14^ | 1•10^-2^ | ^a^ |
| Sema3C-NRP1 + PlxnAi ↔ Sema3C-NRP1-PAi | 1•10^14^ | 1•10^-2^ | ^a^ |
| Sema3C-NRP1 + PlxnD1 ↔ Sema3C-NRP1-PD1 | 1•10^14^ | 1•10^-2^ | ^a^ |
| Sema3C-NRP2 + PlxnAi ↔ Sema3C-NRP2-PAi | 1•10^14^ | 1•10^-2^ | ^a^ |
| Sema3C-NRP2 + PlxnD1 ↔ Sema3C-NRP2-PD1 | 1•10^14^ | 1•10^-2^ | ^a^ |
| Sema3D-NRP1 + PlxnAi ↔ Sema3D-NRP1-PAi | 1•10^14^ | 1•10^-2^ | ^a^ |
| Sema3D-NRP2 + PlxnAi ↔ Sema3D-NRP2-PAi | 1•10^14^ | 1•10^-2^ | ^a^ |
| Sema3F-NRP2 + PlxnAi ↔ Sema3F-NRP2-PAi | 1•10^14^ | 1•10^-2^ | ^a^ |
| Sema3G-NRP2 + PlxnAi ↔ Sema3G-NRP2-PAi | 1•10^14^ | 1•10^-2^ | ^a^ |
| The letter i refers to the four class A Plexins  The letter j refers to the two Neuropilins  ^a^ Assumed diffusion-limited kinetics when no experimental data was available | | | |

**Table S11: Target free ligand concentrations**

| **Protein** | **Molecular Weight (kDa)** | **Target Concentration (pg/mL)** | **Target Concentration (pM)** | **Reference** |
| --- | --- | --- | --- | --- |
| VEGF-A_165_ | 45 | 100 | 2.22 | [9,10,11,13] |
| VEGF-A_121_ | 45 | 100 | 2.22 | [9,10,11,13] |
| sVEGFR1 | 110 | 100 | 0.91 | [[11-13](#_ENREF_11)] |
| PlGF-1 | 56 | 10 | 0.18 | [[5](#_ENREF_5), [14](#_ENREF_14), [15](#_ENREF_15)] |
| PlGF-2 | 64 | 10 | 0.16 | [[5](#_ENREF_5), [14](#_ENREF_14), [15](#_ENREF_15)] |
| VEGF-B_167_ | 42 | 100 | 2.4 | [[16](#_ENREF_16)] |
| VEGF-B_186_ | 60 | 100 | 1.7 | [[16](#_ENREF_16)] |
| VEGF-C | 26 | 1000 | 38 | [[14](#_ENREF_14)] |
| VEGF-D | 26 | 100 | 3.8 | [[17](#_ENREF_17)] |
| Sema3A | 86 | 10000 | 116 | ^a^ |
| Sema3B | 81 | 10000 | 123 | ^b^ |
| Sema3C | 83 | 10000 | 120 | ^b^ |
| Sema3D | 86 | 10000 | 116 | ^b^ |
| Sema3E | 86 | 10000 | 116 | ^b^ |
| Sema3F | 86 | 10000 | 116 | ^b^ |
| Sema3G | 84 | 10000 | 119 | ^b^ |
| ^a^ No cancer-specific measurements available, but [[9](#_ENREF_9)] has healthy measurements  ^b^ Assumed the same order of magnitude as Sema3A | | | | |

**Table S12: Model parameters**

| **Parameter** | **Value** | **Units** | **Reference** |
| --- | --- | --- | --- |
| VEGF-A_165_ secretion rate ^a^ | 0.027 | #/cell/sec | ^b^ |
| VEGF-A_121_ secretion rate ^a^ | 0.013 | #/cell/sec | ^b^ |
| sVEGFR1 secretion rate ^a^ | 0.059 | #/cell/sec | ^b^ |
| PlGF-1 secretion rate ^a^ | 0.00026 | #/cell/sec | ^b^ |
| PlGF-2 secretion rate ^a^ | 0.00011 | #/cell/sec | ^b^ |
| VEGF-B_167_ secretion rate ^a^ | 0.017 | #/cell/sec | ^b^ |
| VEGF-B_186_ secretion rate ^a^ | 0.029 | #/cell/sec | ^b^ |
| VEGF-C secretion rate ^a^ | 0.96 | #/cell/sec | ^b^ |
| VEGF-D secretion rate ^a^ | 0.096 | #/cell/sec | ^b^ |
| Sema3A secretion rate ^a^ | 0.97 | #/cell/sec | ^b^ |
| Sema3B secretion rate ^a^ | 1.50 | #/cell/sec | ^b^ |
| Sema3C secretion rate ^a^ | 1.02 | #/cell/sec | ^b^ |
| Sema3D secretion rate ^a^ | 1.41 | #/cell/sec | ^b^ |
| Sema3E secretion rate ^a^ | 0.0046 | #/cell/sec | ^b^ |
| Sema3F secretion rate ^a^ | 0.52 | #/cell/sec | ^b^ |
| Sema3G secretion rate ^a^ | 0.69 | #/cell/sec | ^b^ |
| Endothelial VEGFR1 | 3,750 | #/cell | [[3](#_ENREF_3)] |
| Endothelial VEGFR2 | 300 | #/cell | [[3](#_ENREF_3)] |
| Endothelial VEGFR3 | 1,000 | #/cell | ^c^ |
| Endothelial NRP1 | 20,000 | #/cell | [[3](#_ENREF_3)] |
| Endothelial NRP2 | 20,000 | #/cell | [[3](#_ENREF_3)] |
| Endothelial PLXNA1 | 1,000 | #/cell | ^c^ |
| Endothelial PLXNA2 | 1,000 | #/cell | ^c^ |
| Endothelial PLXNA3 | 1,000 | #/cell | ^c^ |
| Endothelial PLXNA4 | 1,000 | #/cell | ^c^ |
| Endothelial PLXND1 | 1,000 | #/cell | ^c^ |
| Tumor VEGFR1 ^a^ | 1,100 | #/cell | [[3](#_ENREF_3)] |
| Tumor VEGFR2 ^a^ | 550 | #/cell | [[3](#_ENREF_3)] |
| Tumor VEGFR3 ^a^ | 1,000 | #/cell | ^c^ |
| Tumor NRP1 ^a^ | 39,500 | #/cell | [[3](#_ENREF_3)] |
| Tumor NRP2 ^a^ | 39,500 | #/cell | [[3](#_ENREF_3)] |
| Tumor PLXNA1^a^ | 1,000 | #/cell | ^c^ |
| Tumor PLXNA2^a^ | 1,000 | #/cell | ^c^ |
| Tumor PLXNA3^a^ | 1,000 | #/cell | ^c^ |
| Tumor PLXNA4^a^ | 1,000 | #/cell | ^c^ |
| Tumor PLXND1^a^ | 1,000 | #/cell | ^c^ |
| ECM concentration | 0.75 | μM | [[1](#_ENREF_1)] |
| Receptor internalization rate | 2.8•10^-4^ | sec^-1^ | [[1](#_ENREF_1)] |
| ^a^ These parameters vary based on expression of the corresponding genes.  ^b^ These parameters were tuned to yield the target concentrations in Table S11.  ^C^ No experimental data for Plexin cell surface densities are available, therefore 1,000 was chosen as it falls in the middle of the range of receptor densities. | | | |


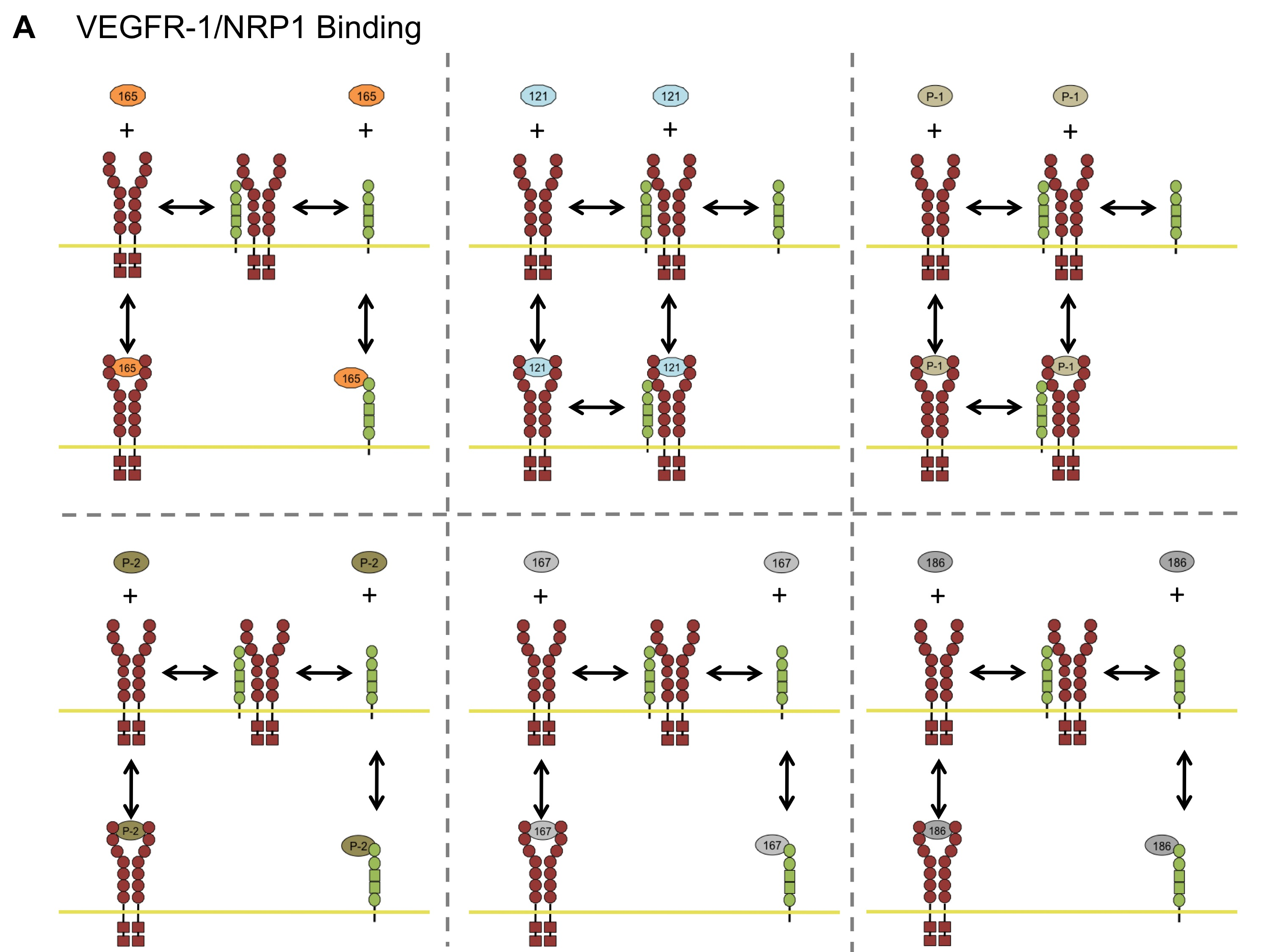


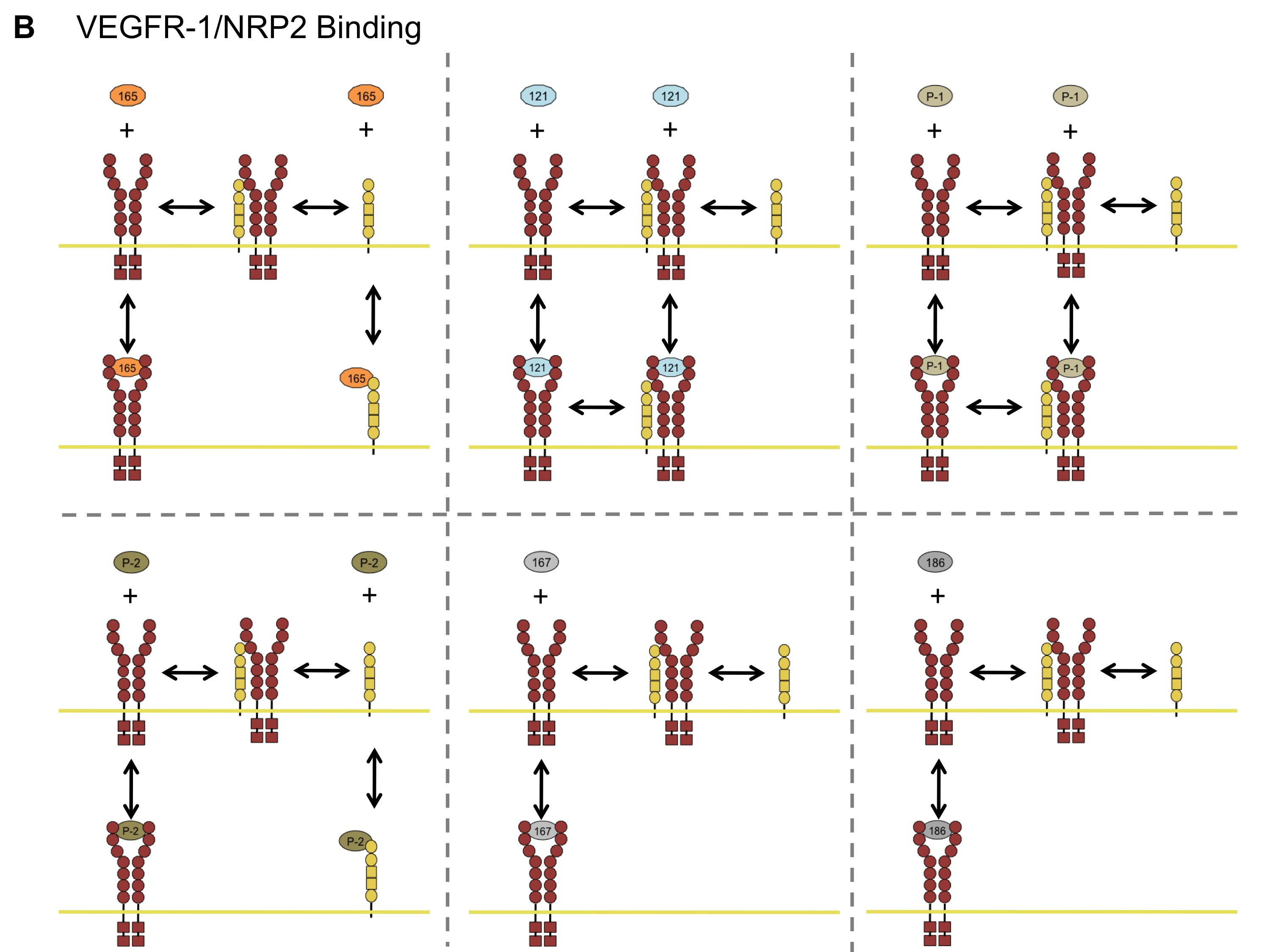
 **Fig.S13**


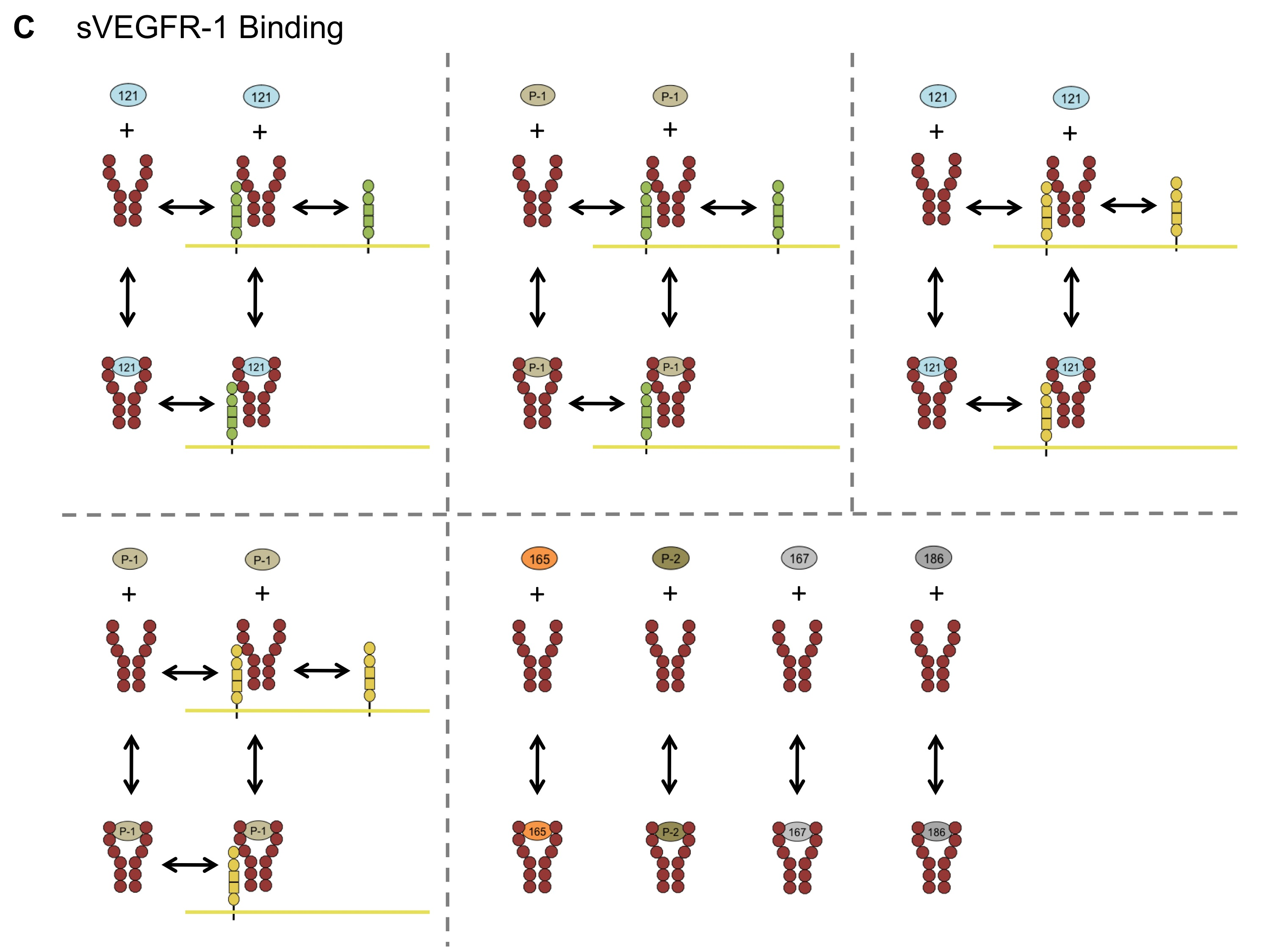


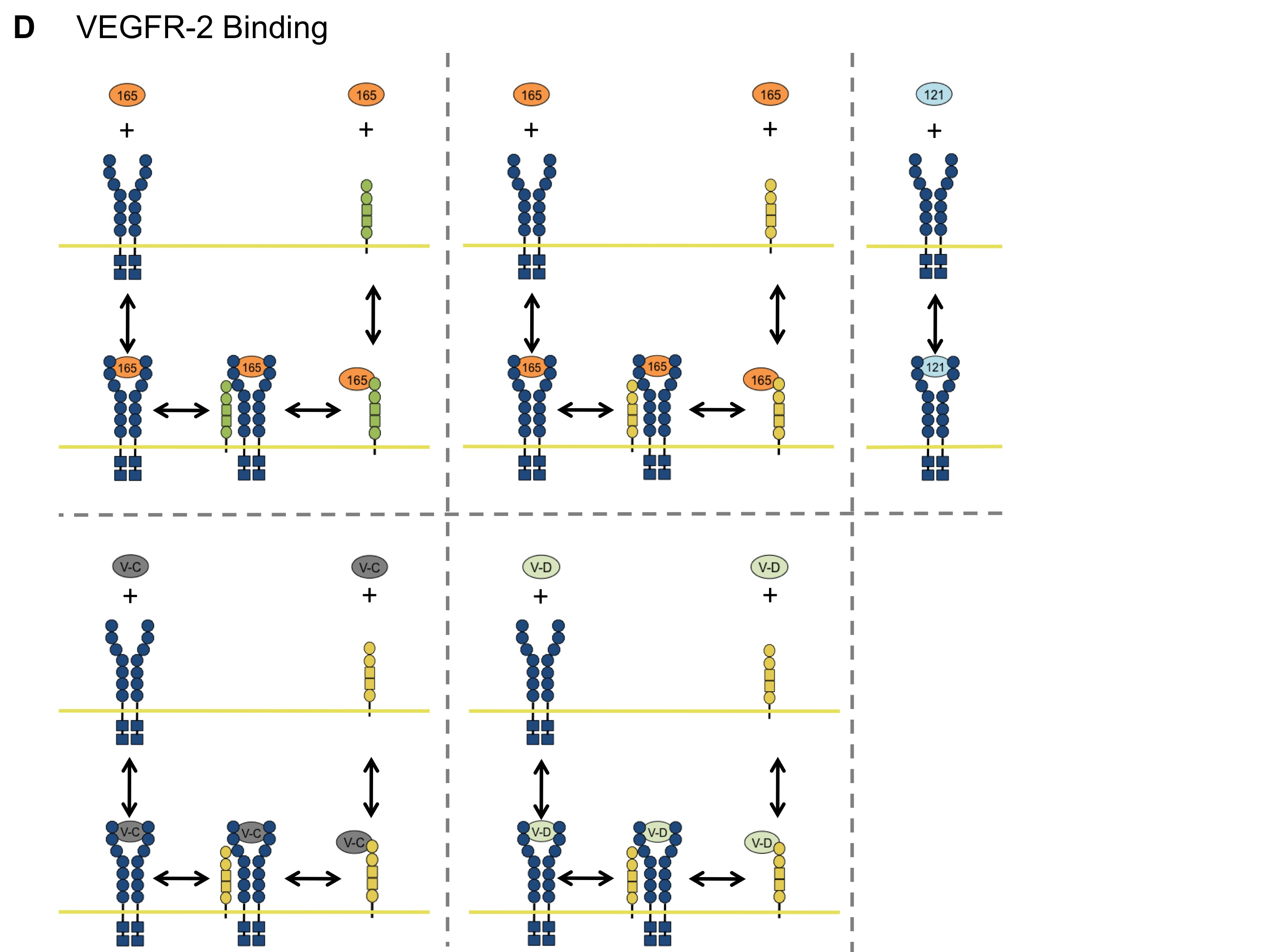
 **Fig.S13 continued**


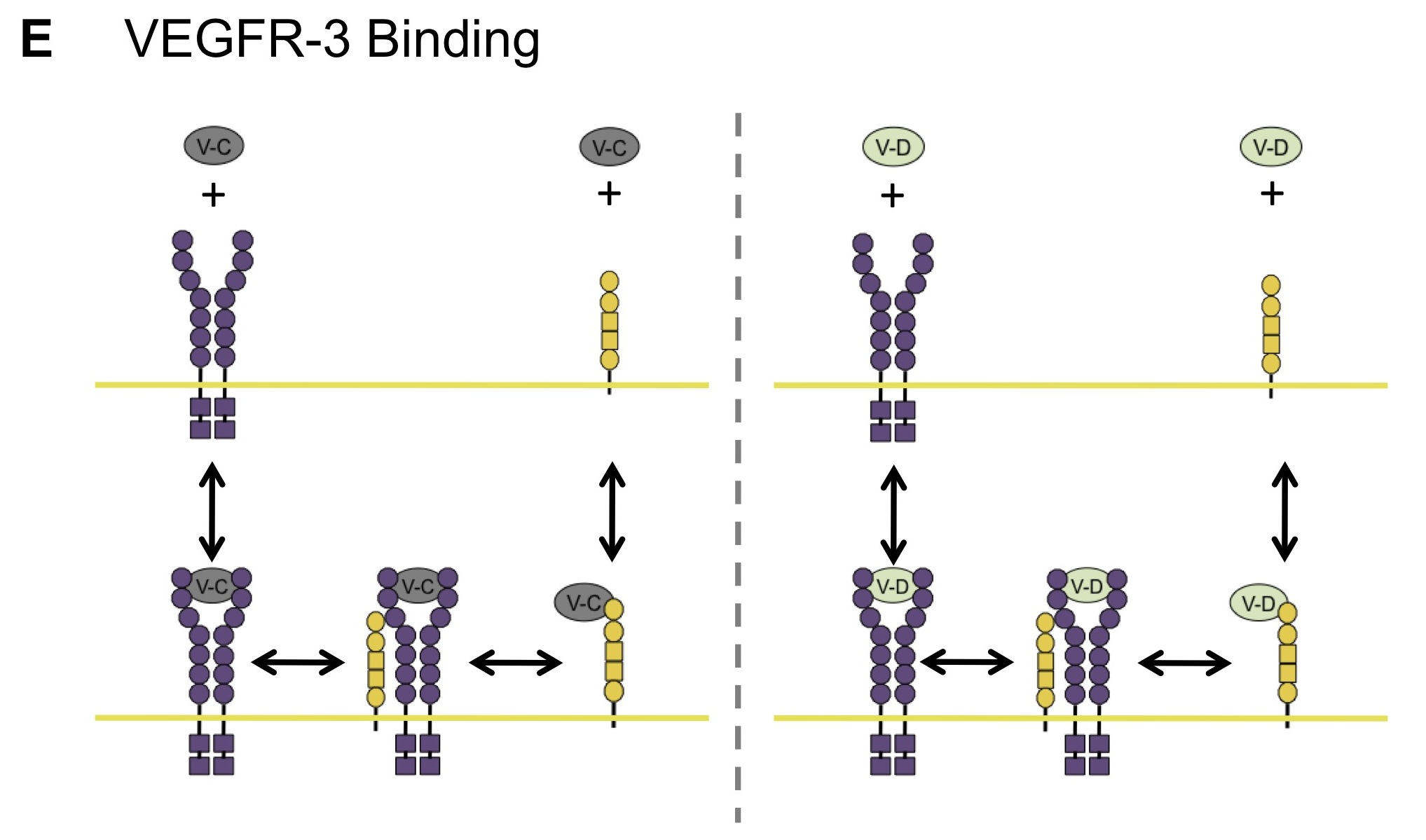


**Figure S13: Reactions in the VEGF/Sema model.** The reactions involving VEGFR-1 (red) and NRP1 (green) (A), VEGFR-1 and NRP-2 (yellow) (B), sVEGFR-1 (C), VEGFR-2 (blue) (D), and VEGFR-3 (purple) (E) are shown with double-sided arrows indicating a reversible reaction takes place.

**
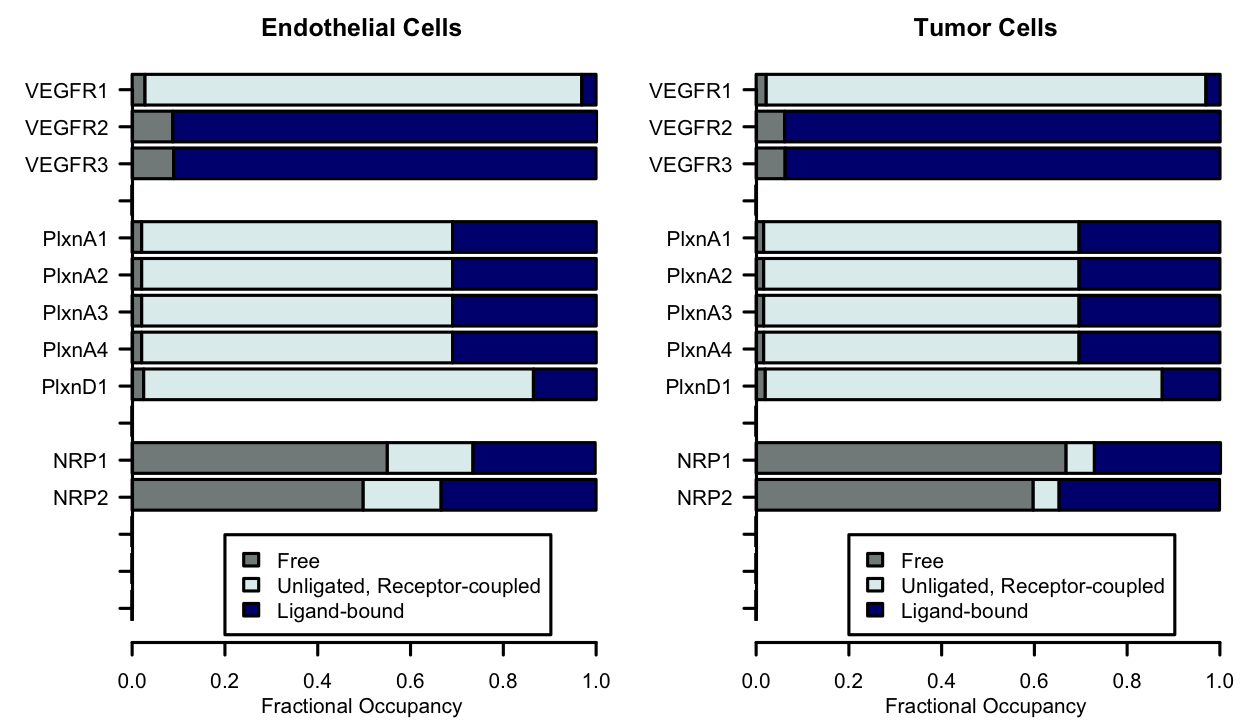
**

**Figure S14: Receptor fractional occupancies at steady-state.** Most VEGFR and Plexin receptors are present in an unligated, Neuropilin-coupled state, with the exception of VEGFR-2 and VEGFR-3, which do not form ligand-independent complexes with Neuropilin.


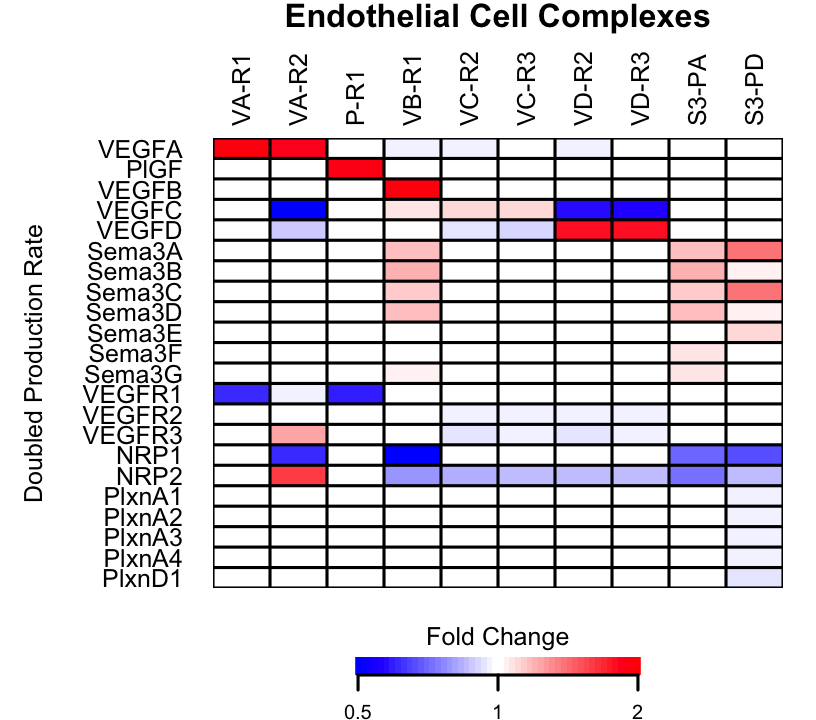


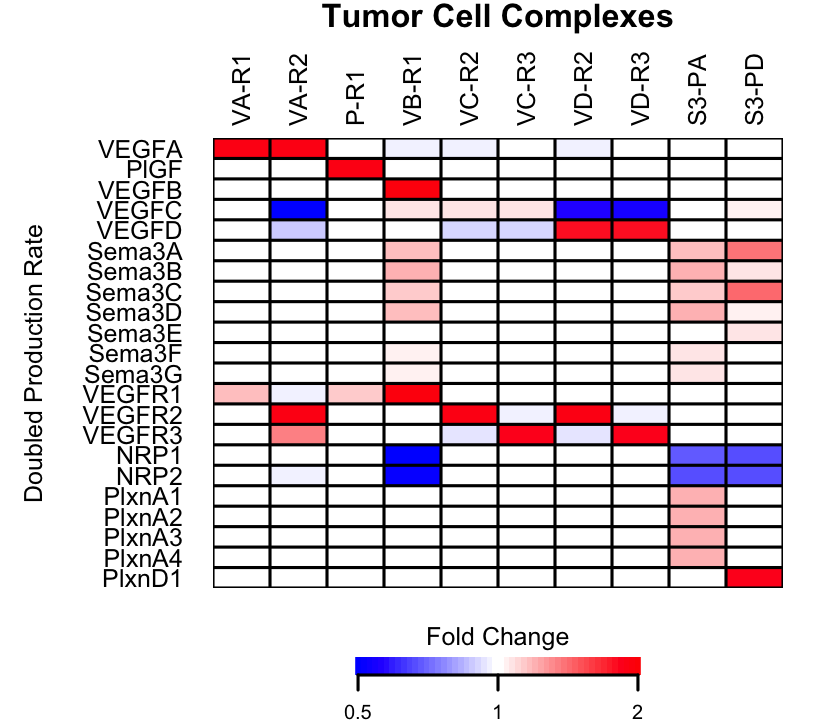


**Figure S15: Effect of ligand secretion rates on receptor binding.** The secretion rate of each of the 16 ligands was doubled while holding other ligand secretion rates steady. The heatmap shows the log2 ratio of the post-doubling to pre-doubling level of each receptor complex. VA-R1 includes all complexes with both VEGF-A and VEGFR-1 (VEGFA_165_-R1, VEGFA_121_-R1, and VEGFA_121_-R1-NRP1), and so on for the rest of the complexes listed along the top of the figure. **SUPPLEMENTAL REFERENCES**

1. Mac Gabhann F, Popel AS: **Targeting neuropilin-1 to inhibit VEGF signaling in cancer: Comparison of therapeutic approaches.** *PLoS Comput Biol* 2006, **2:**e180.

2. Wu FT, Stefanini MO, Mac Gabhann F, Popel AS: **A compartment model of VEGF distribution in humans in the presence of soluble VEGF receptor-1 acting as a ligand trap.** *PLoS One* 2009, **4:**e5108.

3. Finley SD, Engel-Stefanini MO, Imoukhuede PI, Popel AS: **Pharmacokinetics and pharmacodynamics of VEGF-neutralizing antibodies.** *BMC Syst Biol* 2011, **5:**193.

4. Mac Gabhann F, Popel AS: **Interactions of VEGF isoforms with VEGFR-1, VEGFR-2, and neuropilin in vivo: a computational model of human skeletal muscle.** *Am J Physiol Heart Circ Physiol* 2007, **292:**H459-474.

5. Park JE, Chen HH, Winer J, Houck KA, Ferrara N: **Placenta growth factor. Potentiation of vascular endothelial growth factor bioactivity, in vitro and in vivo, and high affinity binding to Flt-1 but not to Flk-1/KDR.** *J Biol Chem* 1994, **269:**25646-25654.

6. Joukov V, Sorsa T, Kumar V, Jeltsch M, Claesson-Welsh L, Cao Y, Saksela O, Kalkkinen N, Alitalo K: **Proteolytic processing regulates receptor specificity and activity of VEGF-C.** *EMBO J* 1997, **16:**3898-3911.

7. Takahashi T, Fournier A, Nakamura F, Wang LH, Murakami Y, Kalb RG, Fujisawa H, Strittmatter SM: **Plexin-neuropilin-1 complexes form functional semaphorin-3A receptors.** *Cell* 1999, **99:**59-69.

8. Gu C, Yoshida Y, Livet J, Reimert DV, Mann F, Merte J, Henderson CE, Jessell TM, Kolodkin AL, Ginty DD: **Semaphorin 3E and plexin-D1 control vascular pattern independently of neuropilins.** *Science* 2005, **307:**265-268.

9. Vadasz Z, Haj T, Halasz K, Rosner I, Slobodin G, Attias D, Kessel A, Kessler O, Neufeld G, Toubi E: **Semaphorin 3A is a marker for disease activity and a potential immunoregulator in systemic lupus erythematosus.** *Arthritis Res Ther* 2012, **14:**R146.

10. Martino MM, Briquez PS, Guc E, Tortelli F, Kilarski WW, Metzger S, Rice JJ, Kuhn GA, Muller R, Swartz MA, Hubbell JA: **Growth factors engineered for super-affinity to the extracellular matrix enhance tissue healing.** *Science* 2014, **343:**885-888.

11. Wu FT, Stefanini MO, Mac Gabhann F, Kontos CD, Annex BH, Popel AS: **A systems biology perspective on sVEGFR1: its biological function, pathogenic role and therapeutic use.** *J Cell Mol Med* 2010, **14:**528-552.

12. Thielemann A, Baszczuk A, Kopczynski Z, Kopczynski P, Grodecka-Gazdecka S: **Clinical usefulness of assessing VEGF and soluble receptors sVEGFR-1 and sVEGFR-2 in women with breast cancer.** *Ann Agric Environ Med* 2013, **20:**293-297.

13. Aoyagi Y, Iinuma H, Horiuchi A, Shimada R, Watanabe T: **Association of plasma VEGF-A, soluble VEGFR-1 and VEGFR-2 levels and clinical response and survival in advanced colorectal cancer patients receiving bevacizumab with modified FOLFOX6.** *Oncol Lett* 2010, **1:**253-259.

14. Rini BI, Michaelson MD, Rosenberg JE, Bukowski RM, Sosman JA, Stadler WM, Hutson TE, Margolin K, Harmon CS, DePrimo SE, et al: **Antitumor activity and biomarker analysis of sunitinib in patients with bevacizumab-refractory metastatic renal cell carcinoma.** *J Clin Oncol* 2008, **26:**3743-3748.

15. Willett CG, Boucher Y, Duda DG, di Tomaso E, Munn LL, Tong RT, Kozin SV, Petit L, Jain RK, Chung DC, et al: **Surrogate markers for antiangiogenic therapy and dose-limiting toxicities for bevacizumab with radiation and chemotherapy: continued experience of a phase I trial in rectal cancer patients.** *J Clin Oncol* 2005, **23:**8136-8139.

16. Li X: **VEGF-B: a thing of beauty.** *Cell Res* 2010, **20:**741-744.

17. Kummel S, Eggemann H, Luftner D, Thomas A, Jeschke S, Zerfel N, Heilmann V, Emons G, Zeiser T, Ulm K, et al: **Changes in the circulating plasma levels of VEGF and VEGF-D after adjuvant chemotherapy in patients with breast cancer and 1 to 3 positive lymph nodes.** *Anticancer Res* 2006, **26:**1719-1726.
